# Overcoming Irinotecan Resistance by Targeting Its Downstream Signaling Pathways in Colon Cancer

**DOI:** 10.3390/cancers16203491

**Published:** 2024-10-15

**Authors:** Shashank Saurav, Sourajeet Karfa, Trung Vu, Zhipeng Liu, Arunima Datta, Upender Manne, Temesgen Samuel, Pran K. Datta

**Affiliations:** 1 Division of Hematology and Oncology, Department of Medicine, UAB Comprehensive Cancer Center, University of Alabama at Birmingham, Birmingham, AL 35233, USA; 2 Birmingham Veterans Affairs Medical Center, Birmingham, AL 35233, USA; 3 Department of Pathology, University of Alabama at Birmingham, Birmingham, AL 35233, USA; 4 Department of Pathobiology, Tuskegee University, Tuskegee, AL 36088, USA

**Keywords:** colorectal cancer, irinotecan, SN38, CDKIs, osteopontin, NF-κB, ISG15, PD-L1, immunomodulation, drug resistance

## Abstract

**Simple Summary:**

Treatments for colorectal cancer (CRC) largely rely on chemotherapeutics. For CRC treatment, irinotecan is widely used in various combinations with other drugs. Despite being an effective drug, irinotecan treatment often leads to drug resistance and tumor relapse. Mechanistically, it inhibits topoisomerase I and induces double-strand DNA breaks, which result in p53-dependent apoptosis. In the current study, we explored irinotecan-mediated, p53-independent pro- and anti-apoptotic effects. Our data show that irinotecan induces cyclin-dependent kinase inhibitors as well as anti-apoptotic proteins [osteopontin (OPN), survivin, and ISG15], the immunomodulatory molecule PD-L1, immune modulation mediated by proliferative NF-κB signaling, and metastatic markers (CD44, Sox2, Oct4, Snail, and Slug). Inhibition of OPN and/or NF-κB signaling potentiates irinotecan efficacy. The combination of OPN and/or an NF-κB inhibitor with irinotecan may lead to increased efficacy with reduced drug resistance.

**Abstract:**

Among the most popular chemotherapeutic agents, irinotecan, regarded as a prodrug belonging to the camptothecin family that inhibits topoisomerase I, is widely used to treat metastatic colorectal cancer (CRC). Although immunotherapy is promising for several cancer types, only microsatellite-instable (~7%) and not microsatellite-stable CRCs are responsive to it. Therefore, it is important to investigate the mechanism of irinotecan function to identify cellular proteins and/or pathways that could be targeted for combination therapy. Here, we have determined the effect of irinotecan treatment on the expression/activation of tumor suppressor genes (including p15^Ink4b^, p21^Cip1^, p27^Kip1^, and p53) and oncogenes (including OPN, IL8, PD-L1, NF-κB, ISG15, Cyclin D1, and c-Myc) using qRT-PCR, Western blotting, immunofluorescence (IF), and RNA sequencing of tumor specimens. We employed stable knockdown, neutralizing antibodies (Abs), and inhibitors of OPN, p53, and NF-κB to establish downstream signaling and sensitivity/resistance to the cytotoxic activities of irinotecan. Suppression of secretory OPN and NF-κB sensitized colon cancer cells to irinotecan. p53 inhibition or knockdown was not sufficient to block or potentiate SN38-regulated signaling, suggesting p53-independent effects. Irinotecan treatment inhibited tumor growth in syngeneic mice. Analyses of allograft tumors from irinotecan-treated mice validated the cell culture results. RNA-seq data suggested that irinotecan-mediated activation of NF-κB signaling modulated immune and inflammatory genes in mice, which may compromise drug efficacy and promote resistance. In sum, these results suggest that, for CRCs, targeting OPN, NF-κB, PD-L1, and/or ISG15 signaling may provide a potential strategy to overcome resistance to irinotecan-based chemotherapy.

## 1. Introduction

In the United States, colorectal cancer (CRC) is the third most prevalent cancer and the second leading cause of cancer death. At diagnosis, more than 50% of CRCs have liver metastases [1]. Chromosomal instability, microsatellite instability, and CpG island methylation are among the genetic and epigenetic alterations that accumulate to cause 70% of CRC cases [2,3]. CRC treatment is primarily dependent on surgical removal followed by standard chemotherapy and radiotherapy. Although these therapies have been used in practice for more than 40 years, results are unsatisfactory due to tumor relapse and drug resistance. For treating CRC, irinotecan (also known as CPT-11) is widely used in combination with other chemotherapy regimens, mainly FOLFIRI [leucovorin, 5-fluorouracil (5-FU), and irinotecan] or FOLFOXIRI (leucovorin, 5-FU, oxaliplatin, and irinotecan) [4,5,6]. The active metabolite of irinotecan, SN38, produced by the enzymatic cleavage of a side chain by the liver enzyme carboxylesterase, is 100–1000 times more potent than irinotecan [7,8]. Irinotecan forms a ternary complex with topoisomerase I (TOPO-I) and DNA and inhibits the TOPO-I-mediated, nicked DNA ligation at replication forks, which induces DNA double-strand breaks, followed by p53-triggered apoptosis [9]. The efficacy of irinotecan has shown to be enhanced by the adenoviral gene-delivery-mediated overexpression of rabbit-carboxylesterase in human colon cancer [10]. Moreover, irinotecan or its metabolite SN38 can be inactivated by uridine diphosphate glucuronosyltransferase 1A1 (UGT1A1), cytochrome P450-3A4 (CYP3A4), and β-glucuronidase [11]. Thus, overcoming the resistance and potentiation of irinotecan efficacy may be desired for future treatment approaches.

Cyclin dependent kinases (CDKs) are regulators of various signaling pathways and contribute to cell division and proliferation along with other kinases, including MAPK and GSK3*β* [12,13,14]. CDKs are negatively regulated by CDK inhibitors (p16^Ink4a^, p15^Ink4b^, p18^Ink4c^, p19^Ink4d^, p21^Cip1^, p27^Kip1^, and p57^Kip2^) in response to DNA damage either by direct inhibition of CDKs or by inhibiting their interaction with substrates (cyclins) [15,16]. In addition to CDK inhibitors (CDKIs), Bax is also involved in the induction of apoptosis upon DNA damage [17]. In contrast, c-Myc, a proto-oncogene, and CyclinD1 (a CDK substrate) induce cell division, growth, and proliferation. Osteopontin (OPN), a secretory glycoprotein, serves as a marker of CRC tumor progression [18]. OPN is involved in the osteoblast to osteoclast conversion and in bone formation and resorption [19,20]. OPN interacts with membrane receptors, integrins, and CD44 and influences cell adhesion and chemotaxis [21,22]. In most CRCs, the levels of OPN are high, and the silencing of this protein leads to inhibition of CRC CMT93 cells. OPN is linked with the *β*-catenin-regulated c-Myc pathway and contributes to cancer progression and metastasis [23,24]. Additionally, for CRC patients, expression of survivin (an anti-apoptotic protein) has also been found to be high, providing stemness and metastatic properties to CRCs [25]. NF-κB signaling, also involved in CRC development and progression, regulates the expression of various pro-inflammatory factors and proto-oncogenes and is present at high levels in irinotecan-treated CRC patients [26]. NF-κB signaling controls the expression and function of immunomodulatory proteins, including interferons, which regulate the function of downstream molecules, including interferon-stimulated gene 15 (ISG15). In CRC tissues, ISG15, a ubiquitin-like molecule, is present at high levels and promotes the migration and proliferation of colon cancer cells; silencing of this protein results in decreased cell proliferation and metastasis. For colon cancer patients, high levels of ISG15 are associated with poor prognosis [27]. For cancer treatment, immunotherapy is emerging as an alternative with fewer off-target effects and better post-therapy quality of life. For this treatment, a hurdle is the deregulation of surface immune markers, including PD-1 and PD-L1. Higher levels of PD-L1 are largely regulated by NF-κB signaling on its transcription as well as its post-transcription stabilization [28]. PD-L1 interacts with the immune inhibitory T-cell receptor PD-1, which is immunosuppressive and induces resistance to immunotherapy [29].

The inefficiency of irinotecan-combined chemotherapeutics and limited immunotherapy for CRC led us to evaluate the cell growth inhibitory and antiapoptotic effects induced by irinotecan and to identify targets for the potentiation of its efficacy. We explored the cytoplasmic expression levels of various pro- and anti-apoptotic proteins, followed by identifying and inhibiting the target proteins or pathways to potentiate the efficacy of SN38. We demonstrate the SN38-mediated, p53-independent growth inhibitory and pro-apoptotic induction of CDKIs and Bax and the cell proliferative activation of OPN, PD-L1, survivin, ISG15, and NF-κB signaling. The silencing of OPN resulted in PD-L1 regulation and the inhibition of NF-κB signaling associated with lower levels of survivin and ISG15. The neutralization of secretory OPN and inhibition of NF-κB signaling also resulted in the decreased survival of CRC cells. Furthermore, RNA sequencing of tumors from irinotecan-treated mice showed up- and downregulation of proliferative/stem markers and inflammatory genes.

## 2. Materials and Methods

### 2.1. Cell Culture

Human colon adenocarcinoma cell lines DLD-1 (ATCC CCL-221, Cellosaurus: CVCL_0248) and SW480 (ATCC CCL-228, Cellosaurus: CVCL_0546) and colon carcinoma cell line RKO (ATCC CRL-2577, Cellosaurus: CVCL_0504) were obtained from ATCC. Mouse adenocarcinoma cell line MC38 (SCC172, Cellosaurus: CVCL_B288) was obtained from Sigma-Aldrich. The FET cell (Cellosaurus: CVCL_A604) line was a kind gift from Dr. Michael Brattain. DLD-1 and SW480 were maintained in McCoy medium; FET, RKO, and MC38 were maintained in RPMI 1640 medium. For generating stable clones with OPN knockdown, DLD-1 cells were transfected with OPN siRNA expressing plasmid pSUPER-OPNi or pSUPER-Control [30]. Cells with stable knockdown were selected with 5 µg/mL of puromycin; single colonies were selected and used for experimentation. For generating stable clones with p53 knockdown, HEK-293T cells were transfected with p53 shRNA expressing plasmid shp53 pLKO.1 puro or pLKO.1-Control [31] to produce lentiviral particles. DLD-1, SW480, and FET cells were incubated with these particles with 8 μg/mL of polybrene for 48 h. Stable knockdown cells were selected with 5 µg/mL, 1.5 µg/mL, and 1 µg/mL of puromycin for DLD-1, SW480, and FET cells, respectively; polyclonal knockdown-stable cells were used for experiments. We used mycoplasma contamination-free cells as determined by LookOut Mycoplasma PCR Detection kits (Sigma-Aldrich, St Louis, MO, USA).

### 2.2. Reagents and Antibodies

Irinotecan and Bay-117082 (Bay) were purchased from Sigma-Aldrich (St Louis, MO, USA). QNZ (EVP4593) and pifithrin HBr (PFT*α*) were purchased from Selleck Chemicals (Houston, TX, USA). SN38 was obtained from Cayman Chemical (Ann Arbor, MI, USA). Abs were purchased as follows: Santa Cruz Biotechnology (Santa Cruz, CA, USA): anti-p21^Cip1^, anti-p27^Kip1^, anti-p65, and anti-p-p65; Cell Signaling (Denver, MA, USA): anti-Bax, anti-c-Myc, anti-Cyclin D1, anti-PARP, anti-p53, anti-PD-L1, anti-survivin, anti-ISG15, and anti-mouse-HRP; Sigma-Aldrich (St Louis, MO, USA): anti-rabbit-HRP; Abcam (Cambridge, UK): anti-OPN; Invitrogen (ThermoFisher Scientific, Waltham, MA, USA): anti-rabbit-AF488 and anti-mouse-AF594.

### 2.3. Immunoblotting

After treatment, cells were lysed with Tris-lysis buffer (50 mM Tris-Cl pH 7.5, 10 mM EDTA, 150 mM NaCl, and 0.5% NP-40). Proteins were resolved with 10% SDS-PAGE and transferred onto PVDF membranes. Proteins were probed with a primary Ab for 3 h at room temperature (RT) followed by a secondary Ab for 1 h at RT. The signals were visualized by enhanced chemiluminescence.

For secretory OPN, conditioned cell growth medium was concentrated using 10 kDa cutoff centrifugal filter units (Sigma-Aldrich, St Louis, MO, USA). Protein contents were normalized with cell numbers and immunoblotted for OPN.

Tumor tissues were minced in chilled RIPA lysis buffer (25 mM Tris pH 7.8, 150 mM NaCl, 1% NP-40, 0.5% sodium deoxycholate and 0.1% SDS) and sonicated for five seconds five times. Lysates were used to determine protein levels by Western blots. Original, uncropped Western blot membrane figures can be found in the Supplementary Materials.

### 2.4. qRT-PCR

Total RNA was isolated from DLD1, SW480, and FET cells using the Trizol reagent according to the manufacturer’s instructions (Invitrogen, Waltham, MA, USA). Total RNA (1 μg) was reverse transcribed with iScript reverse transcriptase super mix (Bio-Rad, Hercules, CA, USA) following the manufacturer’s protocol. This first-strand cDNA was used for qRT-PCR for quantification with Roche SYBR green qPCR master-mix (2×) and normalized with GAPDH. The thermal cycling conditions for quantitative PCR were as follows: 95 °C for 10 min, followed by 30 cycles of 95 °C for 30 s, 55 °C for 30 s, and 72 °C for 30 s. The following qRT-PCR primers were used: p15^Ink4b^- 5′-ATCCCAACGGAGTCAACC-3′ (F), 5′-CTGCCCATCATGACCTG-3′ (R); p21^Cip1^- 5′-CTGCCCAAGCTCTACCTTCC (F), 5′-CCACATGGTCTTCCTCTGCT-3′ (R); p27^Kip1^- 5′-CCGGCTAACTCTGAGGACAC-3′ (F), 5′-TGCAGGTCGCTTCCTTATTC (R); p53- 5′-CCTCAGCATCTTATCCGAGTGG-3′ (F), 5′-TGGATGGTGGTACAGTCAGAGC-3′ (R); IL8- 5′-CATACTCCAAACCTTTCCACCCC-3′ (F), 5′-TCAGCCCTCTTCAAAAACTTC TCCA-3′ (R); CCL3- 5′-ACTTTGAGACGAGCAGCCAGTG-3′ (F), 5′-TTTCTGGACCCACT CCTCACTG-3′ (R); CCL5-5′- CCTGCTGCTTTGCCTACATTGC-3′ (F), 5′-ACACACTTGG CGGTTCTTTCGG-3′ (R); RANKL- 5′-GCCTTTCAAGGAGCTGTGC-3′ (F), 5′-GAGCAAA AGGCTGAGCTTC-3′ (R); GAPDH- 5′-ACCTGCCAAATATGATGAC-3′ (F), and 5′-TCATAC CAGGAAATGAGCTT-3′ (R).

### 2.5. Cell Cytotoxicity Assay

Inhibition of cell viability was determined by use of the MTT [3- (4,5-dimethylthiazol-2-yl)-2,5-diphenyltetrazolium bromide] assay. Cells (4000/well) were seeded in triplicate, treated with drugs as per the experimental requirement, and incubated for 48 h. After treatment, cells were incubated with MTT (0.5 mg/mL) for 3 h followed by lysis using MTT extraction buffer (20% SDS in 50% DMF) on an orbital shaker at 150 rpm for 3 h in the dark. The absorbance was measured at 595 nm using a multi-scanner 96-well plate reader (Agilent, Santa Clara, CA, USA, Bio-Tek, Winooski, VT, USA). Data were normalized with untreated cells and presented as cell survival percentages.

### 2.6. Combinatorial Treatment

Cells were treated with Bay or QNZ for 3 h followed by the addition of various concentrations of SN38. For inhibition of secretory OPN, cells were incubated with 2 μg/mL of OPN Ab in combination with indicated concentrations of SN38.

### 2.7. Immunofluorescence Analysis

Cells were grown and treated on coverslips. After treatment, cells were washed with PBS and fixed with 4% paraformaldehyde for 15 min. Fixed cells were washed with PBS and incubated in 0.5% Triton X-100 to permeabilize the cells. Coverslips were washed again with PBS and incubated with blocking buffer [2% BSA (*w*/*v*) and 0.1% Triton X-100 in PBS] for 1 h followed by three washes with PBS. Coverslips were incubated with an Ab (1:100 ratios in blocking buffer) against respective proteins for 12 h at 4 °C. Coverslips were washed three times with PBS and incubated with either an anti-rabbit or anti-mouse Alexafluor-488 (AF-488)- or Alexafluor-594 (AF-594)-conjugated secondary Ab (1:200 ratios in blocking buffer). Coverslips were again washed thrice with PBS. Coverslips were air-dried and mounted onto glass slides with VectaSheild (DAPI containing) mounting media. Edges were sealed with a transparent enamel solution. Images were acquired with a fluorescent microscope (Keyence, BZ-X series, Osaka, Japan).

For antigen recovery, de-paraffinized and rehydrated tissue sections were incubated in 10 mM citrate buffer (pH 6.0) at boiling temperature for 15 min. After antigen recovery, the above-mentioned steps from permeabilization were followed for staining and imaging.

### 2.8. Reporter Gene Assay

DLD-1, SW480, and FET cells seeded into 12-well plates were transiently co-transfected with CMV-*β*-gal together with pGL2-NF-κB-luciferase [32]. Luciferase activity was normalized with *β*-galactosidase activity and presented as the mean ± S.D. of triplicate measurements.

### 2.9. Tumorigenicity Assay

All animal studies were reviewed and approved by the Institutional Animal Care and Use Committee of the University of Alabama at Birmingham in accordance with international guidelines for biomedical research involving animals. MC38 auto-xenografts were established by subcutaneous injection of 0.75 × 10^5^ cells into the flank regions of C57BL/6 mice (6–7 weeks old). Once tumor sizes reached 150 mm^3^, mice were randomly divided into groups of vehicle control (*n* = 5), irinotecan (5 mg/kg) (*n* = 5), and another dose of irinotecan (15 mg/kg) (*n* = 5). Irinotecan was administered intraperitonially every third day for 24 days. Tumor volumes were calculated by the equation V = L × W^2^ × 0.5, where L is the length and W is the width of a tumor. Growth curves for tumors were plotted as the mean volumes ± S.D. of the tumors of mice from each group.

### 2.10. RNA Sequencing

Total RNA was isolated from tumor tissues in the vehicle control and irinotecan (15 mg/kg) groups. RNA sequencing was performed by Novogene Corporation Inc. (Sacramento, CA, USA) on an Illumina-based NovoSeq PE-150 platform with 6G of raw data per sample.

### 2.11. Statistical Analysis

Results were expressed as the mean ± SEM for three independent experiments. Statistical analysis of the samples was conducted using two-tailed paired Student’s *t*-tests. *p* < 0.05 was considered to be significant.

## 3. Results

### 3.1. SN38 Induces Cell Growth and Apoptotic-Related Expression of Genes in Addition to the Conventional P53 Pathway

**Figure 1:**
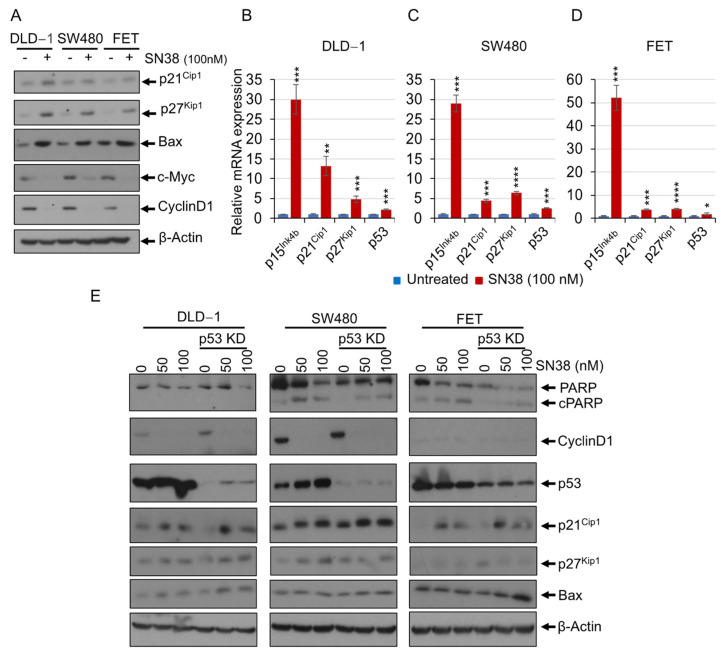
SN38 induces expression of several apoptotic-related genes in addition to the conventional p53 pathway. DLD-1, SW480, and FET cells were treated with SN38 at 100 nM concentration for 48 h. (**A**) Western blots show higher levels of p21^Cip1^, p27^Kip1^, and Bax and lower levels of c-Myc and CyclinD1 upon SN38 treatment. Bar diagrams show the relative mRNA expression of p15^Ink4b^, p21^Cip1^, p27^Kip1^, and p53 in (**B**) DLD-1, (**C**) SW480, and (**D**) FET cells after SN38 treatment. (**E**) p53-silenced DLD-1, SW480, and FET cells were treated with SN38 at 50 or 100 nM concentrations for 48 h. Western blots show the p53-independent, SN38-induced differential levels of p21^Cip1^, p27^Kip1^, and Bax. *β*-Actin served as a loading control. Statistical analysis of the samples was by a Student’s *t*-test. *p* < 0.05 was considered to be significant (* *p* < 0.05, ** *p* < 0.01, *** *p* < 0.001, and **** *p* < 0.001).

SN38 inhibits TOPO-I and induces DNA damage, which in turn recruits p53 and initiates programmed cell death. To identify the molecular mechanisms involved, colorectal adenocarcinoma microsatellite-instable DLD-1 cells and microsatellite-stable cells SW480 and FET were treated with SN38, and several molecules were analyzed for their cellular expression levels. In addition to a p53 increment, SN38 treatment induced p21^Cip1^, p27^Kip1^, and Bax but lowered levels of c-Myc and CyclinD1 (Figure 1A). Moreover, differential gene expression analysis after SN38 treatment resulted in increased p15^Ink4b^ (~30-fold in DLD-1 and SW-480 cells and ~50-fold in FET cells), p21^Cip1^ (~12-fold in DLD-1, ~4-fold in SW-480, and ~3-fold in FET cells), p27^Kip1^ (~5-fold in DLD-1, ~6-fold in SW-480, and ~3-fold in FET cells), and p53 (~2-fold in DLD-1, SW480, and FET) (Figure 1B–D). To delineate the involvement of p53-mediated regulation of alternate pathways, p53 was silenced using lentiviral particles followed by treatment with SN38. p53 silencing did not show any effect on SN38-mediated regulation of p21^Cip1^, p27^Kip1^, Bax, c-Myc, or CyclinD1 but partly restored the cell cytotoxicity as shown by less PARP cleavage compared to parental cells (Figure 1E). Furthermore, inhibition of p53 transactivation with PFT*α* (a p53 inhibitor) did not show any effects on SN38-mediated regulation of p21^Cip1^, p27^Kip1^, or c-Myc (Supplementary Figure S1). SN38-mediated cell cytotoxicity was partially dependent upon p53, and additionally, p15^Ink4b^, p21^Cip1^, p27^Kip1^, Bax, CyclinD1, and c-Myc were involved in its cytotoxicity and efficacy.

### 3.2. SN38 Upregulates Pro-Oncogenic Factors, Including Osteopontin, Survivin, PD-L1, and ISG15

**Figure 2:**
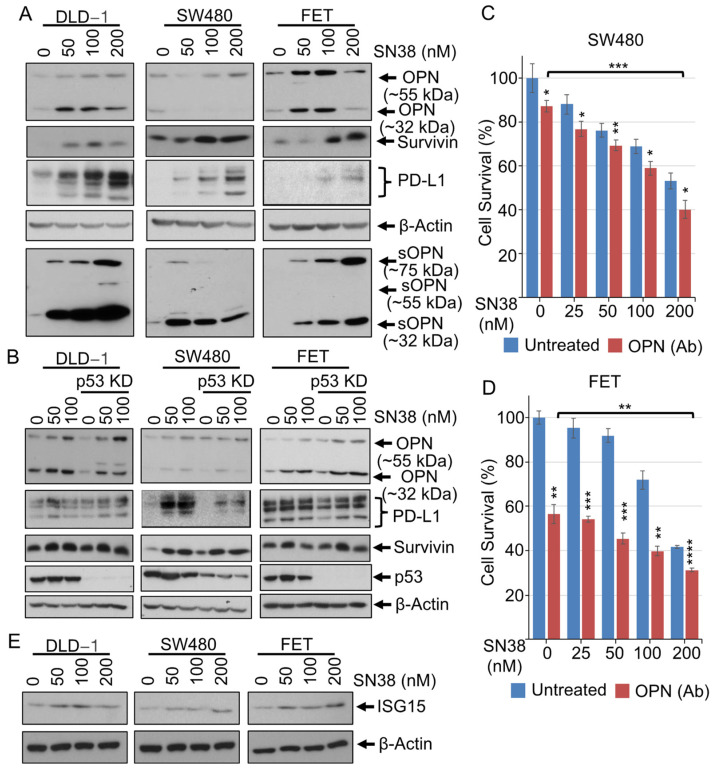
SN38 upregulates pro-oncogenic factors, including survivin, PD-L1, osteopontin, and ISG15. (**A**) DLD-1, SW480, and FET cells were treated with SN38 at increasing concentrations for 48 h. Conditioned media from samples were concentrated and normalized with cell counts and protein concentrations. Western blots show increased cellular levels of OPN, survivin, and PD-L1 and secreted levels of OPN. (**B**) p53-silenced DLD-1, SW480, and FET cells were treated with SN38 at 50 or 100 nM concentrations for 48 h. Western blots show the p53-independent, SN38-induced levels of OPN, PD-L1, and survivin. Cells were treated with an OPN Ab (2 μg/mL) in combination with various concentrations of SN38 for 48 h. Graph showing lower cell survival (%) (MTT assay) of (**C**) SW480 and (**D**) FET cells. (**E**) Western blots showing higher levels of ISG15 after 48 h of SN38 treatment of DLD-1, SW480, and FET cells. *β*-Actin served as a loading control. Statistical analysis of the samples was by a Student’s *t*-test. *p* < 0.05 was considered to be significant (* *p* < 0.05, ** *p* < 0.01, *** *p* < 0.001, and **** *p* < 0.001).

Irinotecan chemotherapy often leads to chemoresistance, immunotherapy resistance, and cancer relapse. To test our hypothesis about SN38, CRC cells DLD-1, SW480, and FET were treated with SN38 at various concentrations for 48 h, and the levels of selected growth-promoting proteins, including cellular and secretory osteopontin (OPN), survivin, and PD-L1 were determined. SN38 treatment induced cellular as well as secretory OPN and increased the immunomodulatory protein PD-L1 and the anti-apoptotic protein survivin (Figure 2A). Since these three cell lines have likely pathogenic missense *TP53* mutations, we checked the effects of SN38 on the wild-type, *TP53*-containing CRC RKO cell line. SN38 treatment showed similar enhancement of OPN, p21^Cip1^, Bax, c-Myc, and survivin (Supplementary Figure S2). Moreover, inhibition or silencing of p53 did not show any effects on the SN38-mediated increases in OPN, survivin, and PD-L1 (Figure 2B). To determine the involvement of secretory OPN in cell proliferation and SN38-mediated inhibition of cell growth, cells were treated with SN38 in combination with an OPN Ab. Although SN38 treatment induced endogenous and secretory OPN in DLD-1 cells, their growth was independent of OPN and unaltered by the inhibition of secretory OPN, whereas SN38 treatment along with an OPN Ab showed a partial reduction in the proliferation of SW480 cells (Figure 2C) and an approximately 50% cell growth inhibition in FET cells (Figure 2D). Moreover, RNA sequencing results for irinotecan-treated MC38 tumors (RNA sequencing results are discussed in the latter part of Section 3) showed more than an 8-fold increase in the growth-promoting protein ISG15. SN38 treatment of DLD-1, SW480, and FET cells also resulted in elevated ISG15 levels (Figure 2E). Thus, the data infer that SN38 induces endogenous and secretory OPN. An anti-apoptotic protein, survivin, and a growth-promoting protein, ISG15, may compromise SN38 efficacy. Cell growth inhibition mediated by the inhibition of secretory OPN points toward a probable target for enhancement of irinotecan efficacy.

### 3.3. SN38 Induces NF-κB Nuclear Localization and Its Activity

**Figure 3:**
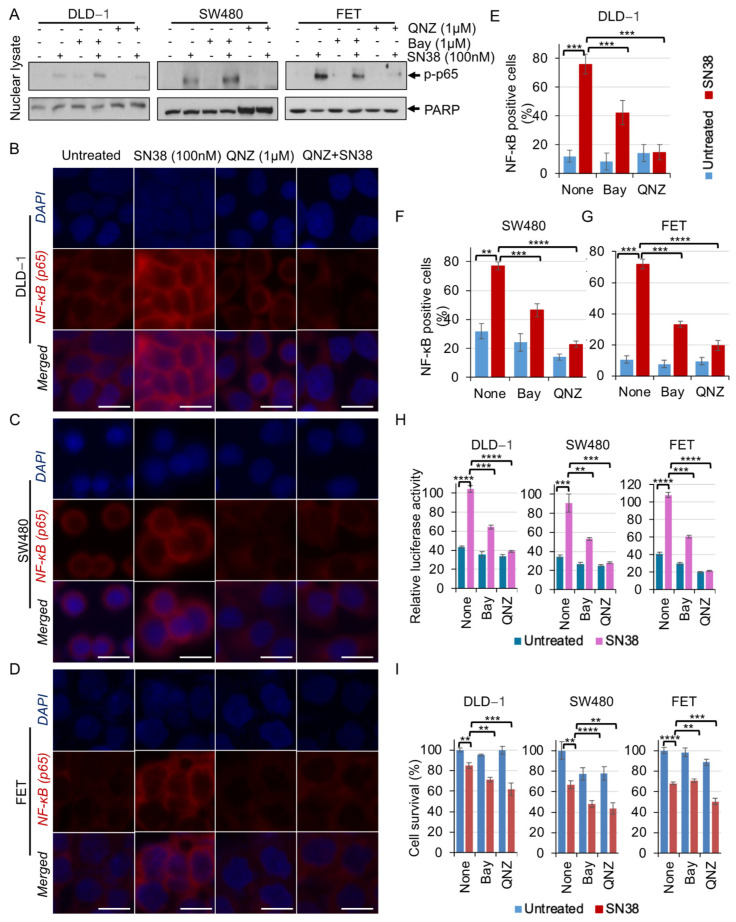
SN38 induces NF-κB activity and its nuclear localization. DLD-1, SW40, and FET cells were pretreated with Bay (1 μM) or QNZ (1 μM) for 3 h followed by in combination with SN38 (100 nM) for 24 h. (**A**) Western blots of nuclear lysates show elevated nuclear localization of p-p65 (NF-κB), an effect inhibited by QNZ. PARP served as a nuclear protein loading control. IF staining of (**B**) DLD-1, (**C**) SW480, and (**D**) FET cells show increased nuclear localization of p-p65 (NF-κB), an effect inhibited by QNZ (scale bar = 10 μm). IF data were analyzed, and NF-κB nuclear-positive cells were counted. Graph showing increased numbers of NF-κB-positive cells (%) (*n* = 1000) in (**E**) DLD-1, (**F**) SW480, and (**G**) FET cells, which were increased by SN38 treatment and partially regulated by Bay and effectively regulated by QNZ. (**H**) DLD-1, SW480, and FET cells were co-transfected with pGL2-NF-κB-luciferase and CMV-*β*-Gal followed by treatment with Bay or QNZ in combination with SN38 under the above conditions. Luciferase activity was measured and normalized with *β*-galactosidase activity. Bar diagram showing the increased relative luciferase activity, which was partially regulated by Bay and effectively regulated by QNZ. (**I**) Bar diagram showing the cell survival (%) (MTT assay) of Bay- or QNZ-treated DLD-1, SW480, and FET cells upon treatment with SN38 for 48 h. Statistical analysis of the samples was by a Student’s *t*-test. *p* < 0.05 was considered to be significant (** *p* < 0.01, *** *p* < 0.001, and **** *p* < 0.001).

For colon cancer cells, the inhibition of NF-κB has been shown to increase the efficacy of SN38 alone or in combination with etoposide and 5-FU [26]. Since most colon cancers have activated NF-κB signaling, which promotes cancer cell stemness and chemoresistance [33,34], modulation of NF-κB activation by SN38 in CRC cells was assayed. Cells were treated with SN38 in the presence of Bay or QNZ (NF-κB inhibitors), and spatial fractionation lysates were used to analyze the differential levels of phospho-p65 in the cytoplasmic and nuclear fractions. SN38 treatment resulted in lower cytoplasmic and higher nuclear NF-κB levels, suggesting the nuclear localization of activated NF-κB, which was rescued by Bay- or QNZ-mediated NF-κB inhibition (Figure 3A). For a proof of concept, SN38-treated cells were subjected to IF, which showed an approximately 75% increased nuclear localization of NF-κB (Figure 3B–D) in DLD-1, SW480, and FET cells. Bay and QNZ inhibited the SN38-induced nuclear localization of NF-κB by 42%, 46%, and 38% and 12%, 21%, and 20%, respectively (Figure 3E–G). Furthermore, NF-κB-luciferase reporter assays showed increased luciferase activity by SN38 treatment, an effect reversed partially by Bay and completely by QNZ (Figure 3H). Additionally, for DLD-1, SW480, and FET cells, inhibition of NF-κB by Bay or QNZ potentiated the SN38 cytotoxicity by approximately 15–20% (Figure 3I). Thus, these results infer that SN38 increases NF-κB nuclear translocation and its activity, and that Bay- or QNZ-mediated inhibition of NF-κB signaling enhances the efficacy of SN38.

### 3.4. SN38 Promotes Immunomodulatory Molecules through Non-Canonical NF-κB Signaling

**Figure 4:**
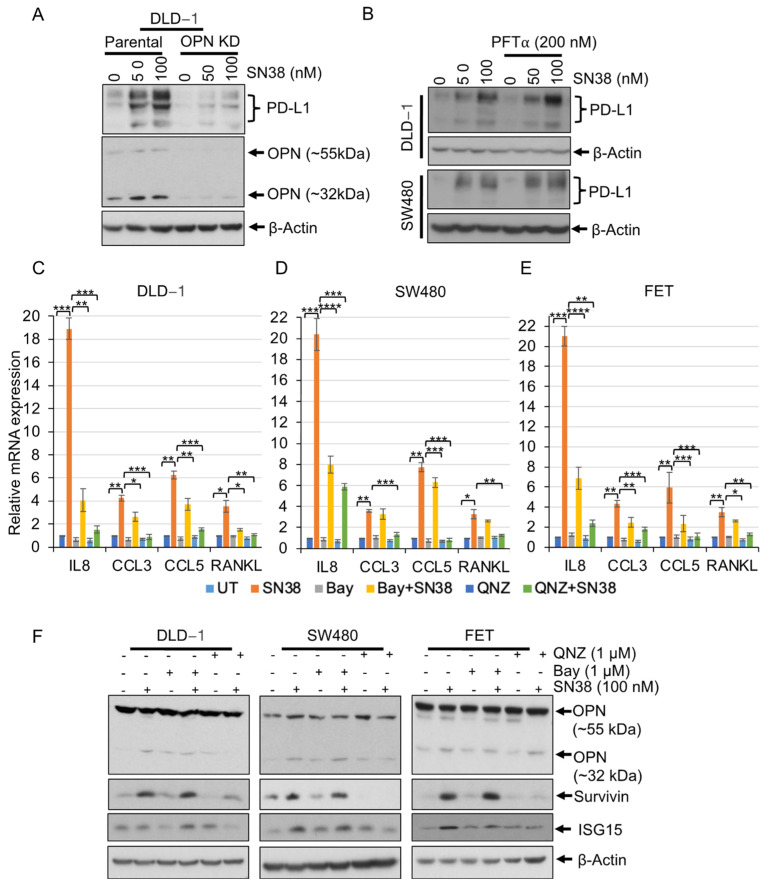
SN38 induces immunomodulatory molecules through non-canonical NF-κB signaling. (**A**) OPN-silenced DLD-1 cells were treated with SN38 at 50 or 100 nM concentrations for 48 h. Western blotting shows OPN silencing decreases the level of PD-L1. (**B**) DLD-1 and SW480 cells were treated with PFT*α* (200 nM) (a p53 transactivation inhibitor) in combination with SN38 for 48 h. Western blots show no effects of p53 inhibition on PD-L1 levels. (**C**) DLD-1, (**D**) SW480, and (**E**) FET cells were pretreated with Bay (1 μM) or QNZ (1 μM) for 3 h followed by in combination with SN38 (100 nM) for 24 h. Bar diagrams showing increased mRNA expressions of IL8, CCL3, CCL5, and RANKL, an effect reduced by QNZ. Relative mRNA expressions of these proteins upon SN38 treatment relative to untreated cells, and the effects of SN38 treatment were compared with SN38 in combination with Bay or QNZ. (**F**) DLD-1, SW480, and FET cells were pretreated with Bay (1 μM) or QNZ (1 μM) for 3 h followed by in combination with SN38 (100 nM) for 48 h. Western blots show inhibition of SN38 on OPN, survivin, and ISG15. *β*-Actin served as a loading control. Statistical analysis of the samples was by a Student’s *t*-test. *p* < 0.05 was considered to be significant (* *p* < 0.05, ** *p* < 0.01, *** *p* < 0.001, and **** *p* < 0.001).

Increased levels of immunomodulatory and inflammatory proteins are associated with drug resistance and cancer cell stemness. PD-L1 has a function in the immune escape of tumors by binding with PD-1 on T lymphocytes and inducing their programmed cell death [35]. SN38 treatment showed an increase in the levels of PD-L1, an effect that was decreased upon OPN silencing (Figure 4A) but unchanged upon p53 inhibition (Figure 4B); OPN silencing did not affect the levels of other proteins. Furthermore, q-RT-PCR data revealed that SN38 treatment resulted in increased IL8, CCL3, CCL5, and TNFSF11 (RANKL) transcription by approximately 20-, 4-, 6-, and 3-fold, respectively, in various CRC cell lines, which was regulated by the inhibition of NF-κB by Bay or QNZ (Figure 4C–E).

Since NF-κB is a central pathway for the regulation of immunomodulatory and inflammatory molecules [26], its involvement was assessed in SN38-mediated OPN, PD-L1, p21^Cip1^, p27^Kip1^, c-Myc, CyclinD1, survivin, ISG15, and Bax proteins. CRC cells treated with SN38 in combination with QNZ showed restricted OPN, survivin, and ISG15 in QNZ-mediated NF-κB inhibition but not with Bay, suggesting the involvement of non-canonical NF-κB signaling (Figure 4F). Collectively, SN38 efficacy is also compromised by NF-κB activation and downstream deregulation of PD-L1, IL8, and CCL3. Additionally, SN38-mediated activation of NF-κB in the regulation of OPN, survivin, and ISG15 was through the non-canonical pathway.

### 3.5. Irinotecan Regulates Tumor Growth by Differential Regulation of Pro- and Anti-Oncogenic Factors

Irinotecan, a widely used chemotherapeutic drug for CRC, inhibits TOPO-I and induces DNA damage [9]. Our in vitro data demonstrated the compromised efficacy of SN38 and its p53-independent role in cell death. To determine the basis of these observations, MC38 cells were xenografted into C57BL6 mice that were treated with irinotecan at 5 or 15 mg/kg doses.

**Figure 5:**
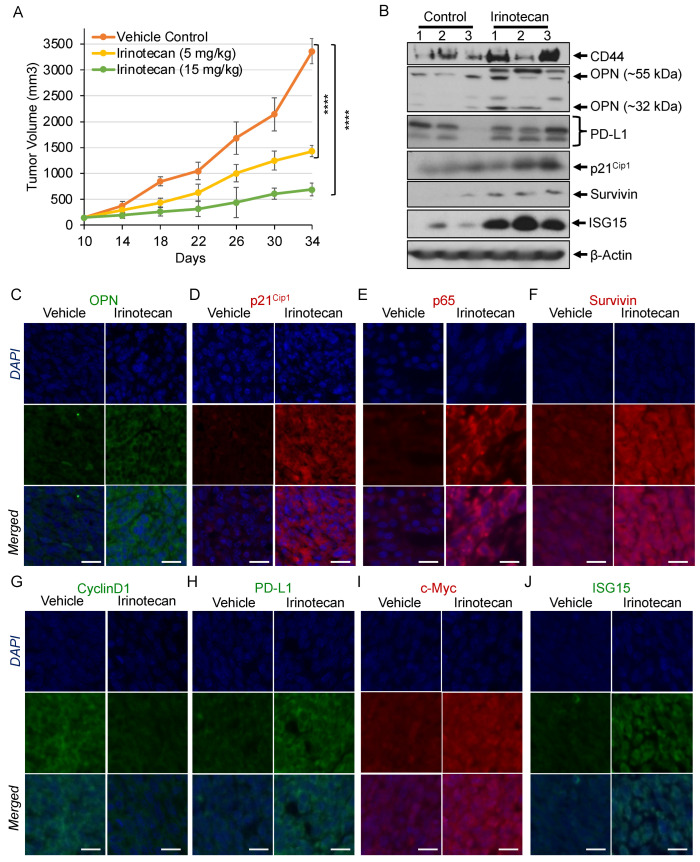
Irinotecan regulates tumor growth by differential regulation of pro- and anti-oncogenic factors. MC38 tumor-bearing mice were treated every third day with irinotecan (5 mg/kg) (*n* = 5) or irinotecan (15 mg/kg) (*n* = 5) for 24 days. Tumor volumes were calculated by the equation V = L × W^2^ × 0.5, where L is the length and W is the width of a tumor. (**A**) Graph showing the kinetics of tumor growth. (**B**) Western blotting showing high levels of CD44, OPN, PD-L1, p21^Cip1^, survivin, and ISG15 in tumor lysates after treatment of tumor-bearing mice with irinotecan (15 mg/kg). IF analysis showing increased (**C**) OPN, (**D**) p21^Cip1^, (**E**) p65, (**F**) survivin, (**G**) PD-L1, (**H**) c-Myc, and (**I**) ISG15 and (**J**) decreased CyclinD1 in the tumor tissues of mice after treatment with irinotecan (15 mg/kg) (scale bar = 10 μm). *β*-Actin served as a loading control. Statistical analysis of the samples was by a Student’s *t*-test. *p* < 0.05 was considered to be significant (**** *p* < 0.001).

Tumor growth kinetics showed that irinotecan reduced average tumor growth to 1428 mm^3^ and 691 mm^3^ at 5 and 15 mg/kg doses, respectively, whereas the vehicle-treated group reached 3363 mm^3^ (Figure 5A) through the course of the treatment. Western blot analysis of tumor tissue lysates showed elevated OPN, PD-L1, p21^Cip1^, c-Myc, survivin, CD44, and ISG15 upon irinotecan treatment (Figure 5B). Furthermore, IF staining of irinotecan-treated tumor tissue sections showed elevated OPN, PD-L1, p21^Cip1^, c-Myc, p65 (NF-κB), ISG15, and survivin, along with lower CyclinD1 compared to the untreated group (Figure 5C–J). Thus, in vivo irinotecan treatment validates the in vitro data in regulating growth-related proteins, and its efficacy may be potentiated by regulating or inhibiting OPN, PD-L1, and NF-κB.

### 3.6. Irinotecan Treatment Regulates Immune and Inflammatory Genes in Mice

**Figure 6:**
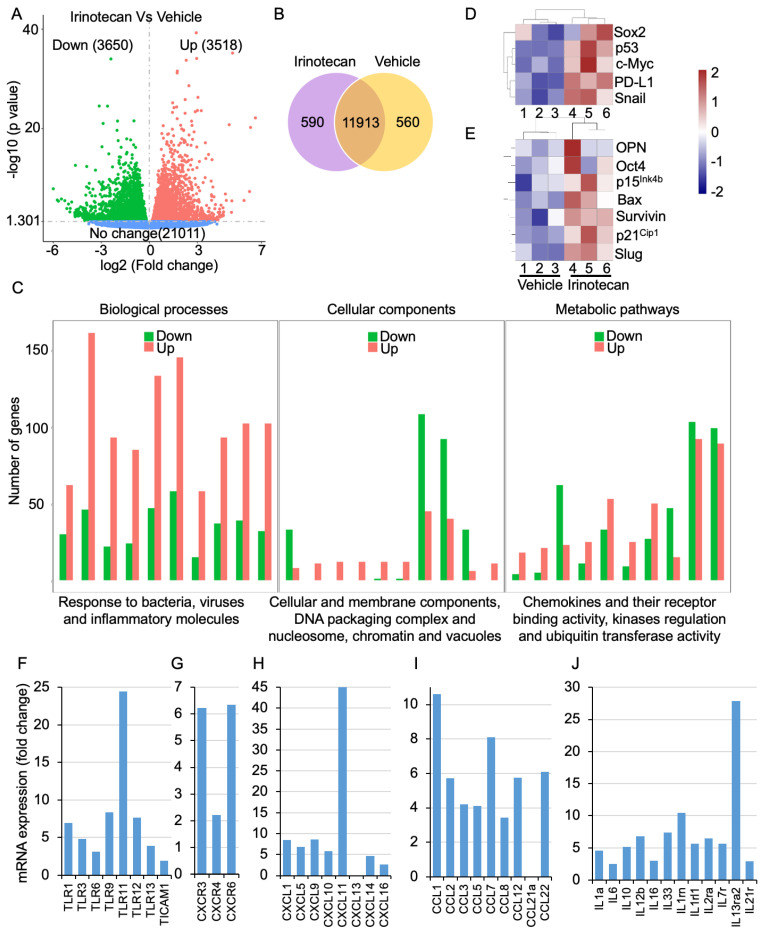
Irinotecan treatment regulates immune and inflammatory genes. RNA sequencing analysis after irinotecan (15 mg/kg) treatment compared to the vehicle control group (**A**) showing the expression of 3518 upregulated and 3650 downregulated genes. (**B**) Venn diagram showing the expression of 590 distinctive genes after irinotecan treatment and 560 distinctive genes in the vehicle control group. (**C**) GO pathway analysis showing the differential expression of various genes involved in biological processes, cellular components, and metabolic pathways. (**D**,**E**) Heatmaps showing high expressions of Sox2, p53, c-Myc, PD-L1, Snail, OPN, Oct4, p15^Ink4b^, Bax, survivin, p21^Cip1^, and Slug after treatment with irinotecan. Bar diagram showing mRNA expression from RNA seq data (**F**) increased TLR, (**G**) CXCR, (**H**) CXCL, (**I**) CCL, and (**J**) interleukins and their receptors (*p* < 0.01).

Since irinotecan induces p65 phosphorylation and nuclear localization, we tested the effects of irinotecan on MC38 xenografted tumors by analysis of differential gene expression using RNA sequencing. Irinotecan treatment resulted in 3518 upregulated and 3650 downregulated genes (Figure 6A) with the expression of 590 and 560 unique genes (Figure 6B) in the treatment and control groups, respectively. Irinotecan treatment resulted in a myriad of differentially expressed genes involved in biological processes, cellular components, and molecular functions (Figure 6C). These genes are mostly involved in elevated immune response, in inflammation, and in regulation of the MEK-ERK and JAK-STAT cascades (Supplementary Figure S3). Furthermore, RNA sequencing analysis of specific genes involved in apoptosis and growth showed elevated p15^Ink4b^, p21^Cip1^, p53, Bax, Sox2, Snail, Slug, PD-L1, c-Myc, survivin, OPN, and Oct4 (Figure 6D,E). Irinotecan treatment also resulted in differential transcript expression of CRC-related genes, including those for collagen, claudin1, growth differentiation factor 15 (Gdf15), p49^NF-κB2^, TFL, ALS, and Bcl2l14 (Supplementary Figure S4).

Since in vitro data suggested the SN38-mediated upregulation of NF-κB, expressions of its downstream immune-responsive and inflammatory genes were analyzed. Irinotecan treatment resulted in a more than 3-fold increase in transcripts of TLR-1, -3, -9, -11, -12, and -13 and an approximately 2-fold increase in TLR-6 and TLR adaptor molecule 1 (TICAM1) (Figure 6F). Additionally, upon irinotecan treatment, C-C motif chemokine ligands (CCLs) CCL-1, -2, -5, -7, -8, -12, and -22 were increased by more than 3-fold, whereas CCL-21A transcripts were reduced (Figure 6G). Moreover, C-X-C motif chemokine receptors (CXCRs) and ligands (CXCLs) were also increased. C-X-C motif chemokine receptors CXCR-3 and -8 were increased by more than 3-fold and CXCR-4 by 2-fold (Figure 6H). Irinotecan treatment showed increased transcripts for C-X-C motif chemokine ligands CXCL-1, -5, -9, -10, -11, -14, and -16 by 3-fold and decreased expression of CXCL-13 (Figure 6I). Since NF-κB activation also regulates interleukins, irinotecan treatment resulted in at least a 3-fold increase in IL-1a, -6, -10, -12b, -16, and -33 and interleukin receptors IL-1rn, -1rl1, -2ra, -7r, and -13ra2 (Figure 6J). Altogether, irinotecan treatment of MC38 syngeneic tumors showed NF-κB activation and regulation of target inflammatory and immune-related genes, which may compromise drug effectiveness and sensitivity.

## 4. Discussion

Irinotecan is a camptothecin-derived chemotherapeutic drug widely used for the treatment of CRC and other cancers. Liver carboxylesterase-mediated hydrolysis of irinotecan converts it into the bioactive compound SN38 [7]. SN38 inhibits TOPO-I and forms a TOPO-I/DNA/SN38 ternary complex, resulting in irreversible double-strand DNA damage, which leads to p53 recruitment and subsequently to apoptosis. Although irinotecan is an inhibitor of TOPO-I, it also induces reactive oxygen species and activates the JNK and p38-MAPK pathways [36]. Irinotecan and its metabolite SN38 interact with the p53 inhibitor E3-ubiquitin ligase MDM2 and the anti-apoptotic protein Bcl-xl to induce TOPO-I-independent apoptosis [26,37]. Given the above-mentioned facts, irinotecan is a widely used second-line chemotherapeutic drug that has been used in combination with other drugs for CRC or other solid cancer for more than three decades. There are several reports suggesting irinotecan-mediated toxicity, neutropenia, and drug resistance compromises the efficacy of the drug [38,39]. Here, we explored TOPO-I/p53-independent mechanisms that are involved in decreasing irinotecan efficacy and could be targeted to potentiate the effects of therapeutics in the future.

CRC persistence, growth, and metastasis are dependent on various signaling molecules and pathways. Since TOPO-I inhibition and p53 recruitment are a major axis for irinotecan-mediated cytotoxicity, we explored other dimensions and found the p53-independent regulation of various pro- and anti-apoptotic markers. SN38 treatment resulted in increased p15^Ink4b^, p21^Cip1^, p27^Kip1^, and Bax and decreased c-Myc and CyclinD1. Additionally, p53 silencing (p53-shRNA) or its inhibition by PFT*α* did not change the effect of SN38 (Figure 1). Furthermore, SN38 treatment caused increases in various growth-promoting proteins, including OPN. A bone sialoprotein secreted from osteoblasts and osteoclasts, OPN is highly expressed in bone lesions such as those of rheumatoid arthritis. It is also involved in tumor progression and metastasis [40,41,42]. In DLD-1, SW480, and FET cells, SN38 showed increased endogenous and secretory OPN. Moreover, the inhibition of secretory OPN by an Ab resulted in restricted cell growth and caused increases in the cytotoxicity of SN38, whereas OPN silencing did not affect cell survival or cytotoxicity. The levels of PD-L1 were downregulated by OPN silencing, suggesting that it is involved in OPN signaling in CRC growth and that it is a potential target for treatment. In addition, surgical removal of CRCs is challenging because of their location, and treatment generally depends on chemotherapy. Adjuvant immunotherapy could be helpful, but we and others have found that irinotecan or SN38 treatment alters PD-L1 expression, diminishing the probability of useful combinatorial immunotherapy [43]. SN38 treatment also increases levels of the pro-survival protein, survivin, and the growth-promoting protein ubiquitin-like protein, ISG15 (Figure 2). ISG15 has been linked to a poor CRC prognosis and is a novel molecular target for therapy [44,45,46]. High levels of ISG15 have been shown in various CRC cell lines and patient samples, and it attenuates the ubiquitin/proteasome pathway by interfering with protein polyubiquitination/degradation. In CRCs, this results in the abnormal degradation of proteins involved in tumorigenesis, and high levels of ISG15 are linked to a poor prognosis [27,47]. An SN38-mediated increase in ISG15 may serve as a target for the potentiation of irinotecan efficacy.

Most CRCs have high NF-κB activity, a proliferative and inflammatory gene-regulating pathway, which helps cancer cells survive and increases drug resistance. It interferes with several chemotherapeutic drugs by increasing anti-apoptotic and pro-survival genes. For HT29 cells, inhibition of NF-κB by AS602868 (an IKK2 kinase inhibitor) improves, by 30–50%, the growth inhibitory effect of irinotecan [26]. Not only is there inherently higher NF-κB activity in CRCs, SN38 also induces its activation and nuclear localization. Its inhibition by Bay or QNZ resulted in the potentiation of SN38 efficacy (Figure 3). Additionally, for LoVo colon cancer cells, irinotecan inhibits mTOR and activates the autophagy-related genes, *ULK1*, *ATG13*, *ATG4L*, and *AMBRA1*, promoting autophagy with increased numbers of lysosomes and lysosomal activity, which contribute to irinotecan resistance [39]. Similarly, upon SN38 treatment of cells, there is higher OPN, PD-L1, survivin, and NF-κB activity, which decreases drug efficacy (Figure 1, Figure 2 and Figure 3). Moreover, QNZ-mediated NF-κB inhibition in irinotecan-resistant LoVo cells shows dose-dependent inhibition of cell survival and reduced autophagic and metastatic markers [48]. Likewise, SN38 treatment increases mRNA levels of immunomodulatory signaling molecules IL8, CCL3, and CCL5, which are reduced by NF-κB inhibition. RANKL, an osteoprotegerin ligand (OPGL) and a transmembrane glycoprotein belonging to the TNF cytokine family, induces NF-κB through RANK (receptor activator nuclear factor); it is upregulated in 75% of CRCs [49,50,51,52]. Its levels correlate with a poor prognosis for CRC, and blockage of the RANKL-RANK signaling inhibits CRC growth and the bone resorption caused by CRC [53,54,55]. SN38 treatment results in increased RANKL, an effect rescued by Bay- or QNZ-mediated inhibition of NF-κB. Additionally, QNZ-mediated NF-κB inhibition showed a reduction in SN38-induced OPN, ISG15, and survivin, whereas Bay-mediated NF-κB inhibition did not alter OPN or survivin, suggesting the involvement of the non-canonical NF-κB pathway in OPN, ISG15, and survivin upregulation (Figure 4). The NF-κB pathway is involved in the positive regulation of inflammatory and proliferative genes, which reduces the efficacy of irinotecan and SN38 as well as immunotherapy. Thus, the targeting of NF-κB, OPN, and RNAKL pathways to enhance irinotecan efficacy may open a new avenue for developing chemo- and immunotherapy combinations.

Irinotecan in combination with 5-FU and oxaliplatin is widely used for the treatment of CRC, but it causes toxicity with severe side effects and sometimes drug resistance and cancer relapse [39,56,57]. Our data also showed that irinotecan inhibited tumor growth, but it upregulated the oncogenic molecule OPN and activated the proliferative factor NF-κB. Additionally, an increase in survivin and CD44 expression showed its involvement in stemness and metastatic properties, which could be the basis of drug resistance and tumor relapse (Figure 5). Furthermore, increases in PD-L1 and upregulation of inflammatory genes may provide another approach for combined chemo- and immunotherapy.

Irinotecan induces the differential expression of more than 7000 genes that are involved in various biological processes and molecular functions. Additionally, the increased expressions of Sox2, Snail, Slug, and Oct4 by irinotecan may be the basis for increased stemness and metastasis, thus supporting drug resistance and relapse in response to therapy. Moreover, the irinotecan-induced NF-κB pathway and the expression of inflammatory genes, including TNFs, interleukins, and chemokines, may contribute to resistance and chemotherapeutic-related ailments.

Thus, targeting the NF-κB pathway in combination with irinotecan treatment may be considered. In addition to the modulation of apoptotic, proliferative, and inflammatory genes, irinotecan treatment also affected the expression of several membrane ion-exchange channel proteins and receptors, including XCT, GPCR8, Claudin1, and CD163. Irinotecan treatment also caused an increase in the expression of mitochondrial genes, including ND-4 and -5 and mitochondrial cytochrome B (Supplementary Figure S4). Irinotecan-mediated regulation of membrane and mitochondrial proteins may lead to research relating to minimizing the resistance to this drug.

Additionally, an increased level of YAP (Yes-associated protein) and Hippo signaling also regulate CRC-invasion and -metastasis [58]. Targeting Hippo signaling in combination with the KRAS inhibitor showed an increased efficacy of the inhibitor on “undruggable” KRAS-inhibitor-resistant CRC, suggesting the involvement of Hippo signaling in chemoresistance [59]. Moreover, upregulated Hippo signaling showed an increased level of secretory OPN in bone-marrow-derived macrophages [60] and was also found to increase co-upregulation in transitional cell carcinoma [61]. Thus, targeting OPN and the Hippo pathway might emerge as a promising avenue for drug sensitization in CRC treatment.

## 5. Conclusions

**Figure 7:**
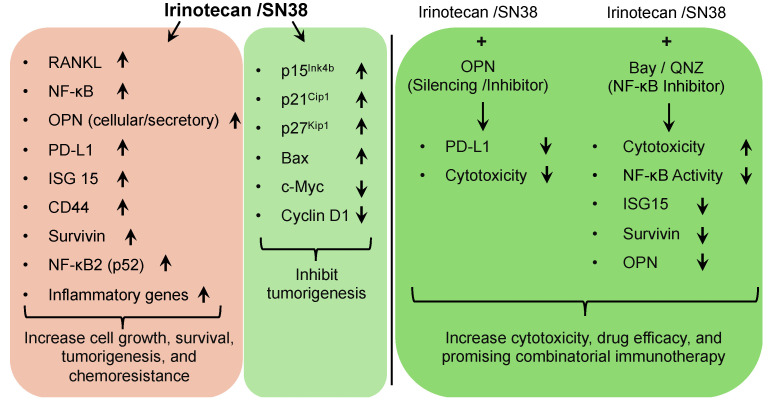
Graphical summary: p53-independent effects of irinotecan in colorectal cancer. ↑ Denotes upregulation and ↓ denotes downregulation of protein expression or signaling.

Although, for decades, irinotecan has been useful as a pro-drug for treatment of CRCs, treatment-related ailments and drawbacks have been neglected, which led us to investigate its effects. Our data suggest that irinotecan upregulates various oncogenes, proliferative pathways, and metastatic markers, which may compromise its efficacy. SN38 induces p53-independent CDKIs and regulates cancer cell growth. OPN silencing regulates the SN38-mediated increase in PD-L1. Inhibition of non-canonical NF-κB signaling by QNZ results in the regulation of SN38-induced survivin and ISG15 (Figure 7). The targeting of OPN, PD-L1, ISG15, and NF-κB pathways may elevate irinotecan potency and lead to its combination with immunomodulatory therapies for CRC prognostic strategies.

## Data Availability

The original contributions presented in the study are included in the article/Supplementary Materials; inquiries can be directed to the corresponding author. The raw data supporting the conclusions of this article will be made available by the corresponding author on request.

## Supplementary Materials

The following supporting information can be downloaded at https://www.mdpi.com/article/10.3390/cancers16203491/s1, Figure S1: SN38-mediated regulation of p21^Cip1^, p27^Kip1^, and c-Myc is independent of p53 inhibition; Figure S2: SN38-mediated effects on wt p53-RKO cells; Figure S3: GO analyses of RNA sequencing data analysis of irinotecan-treated MC38 tumors; Figure S4: Heatmap of RNA sequencing analysis of irinotecan-treated MC38 tumors. Original, uncropped Western blot images.
