# Supplementary material for: Overcoming Irinotecan Resistance by Targeting Its Downstream Signaling Pathways in Colon Cancer
Source: Cancers (Basel). 2024 Oct 15;16(20):3491. doi: 10.3390/cancers16203491 (PMC11505920; doi:10.3390/cancers16203491)

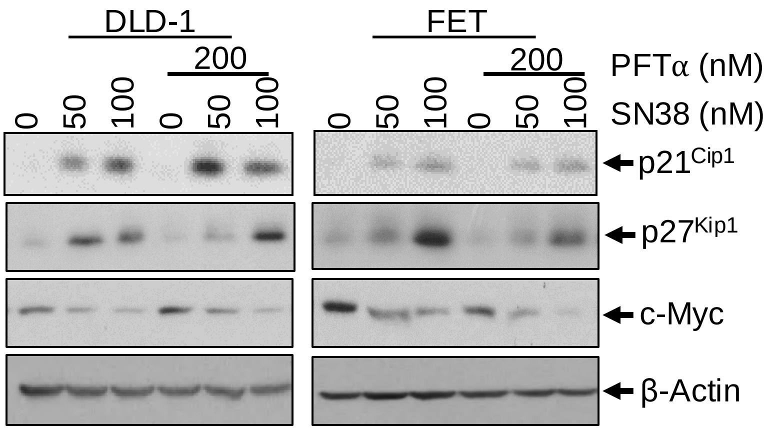

Supplementary Figure S1. SN38-mediated regulation of $p21^{Cip1}$, $p27^{Kip1}$ and c-Myc is independent of p53 inhibition. DLD-1 and FET cells were treated with SN38 at 50 or 100 nM concentrations in combination with PFTα (a p53 transactivation inhibitor) (200 nM) for 48 h. Western blots show the p53-independent, SN38-induced differential levels of $p21^{Cip1}$, $p27^{Kip1}$, and c-Myc. β-Actin served as loading control.

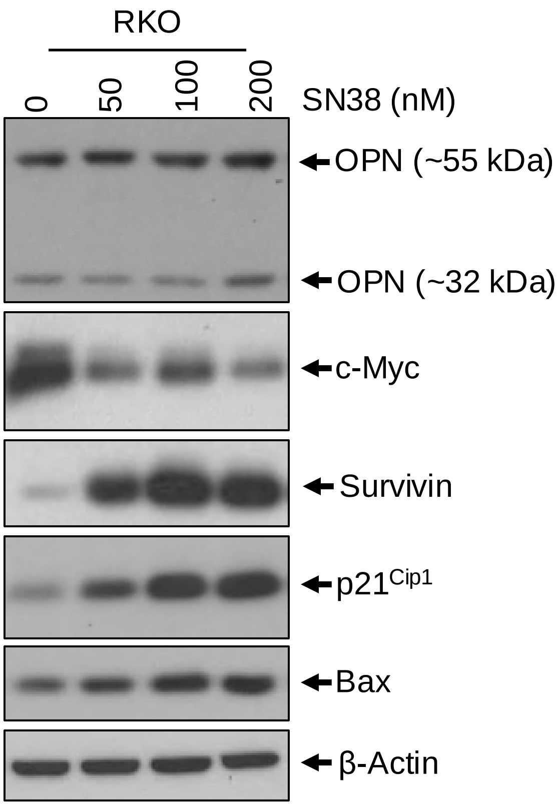

Supplementary Figure S2. SN38-mediated effects on wt p53-RKO cells. RKO cells were treated with various concentrations of SN38 for 48 h. Western blots show increased cellular levels of OPN, survivin, p21^{Cip1}, and Bax and a decreased level of c-Myc. β-Actin served as loading control.

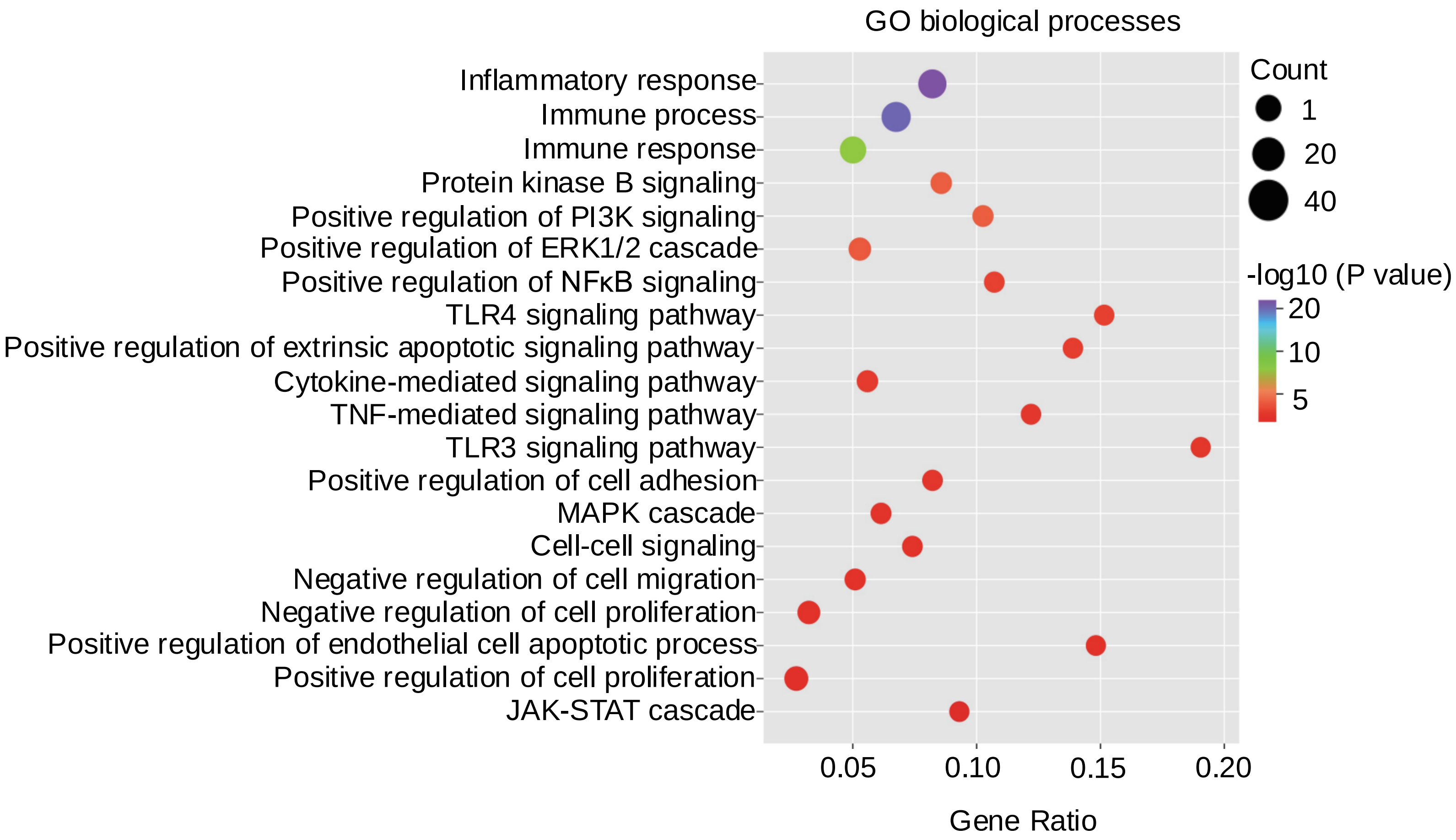

Supplementary Figure S3. GO analyses of RNA sequencing data analysis of irinotecan-treated MC38 tumors. Graphical representation of differentially expressed genes involved in GO biological processes.

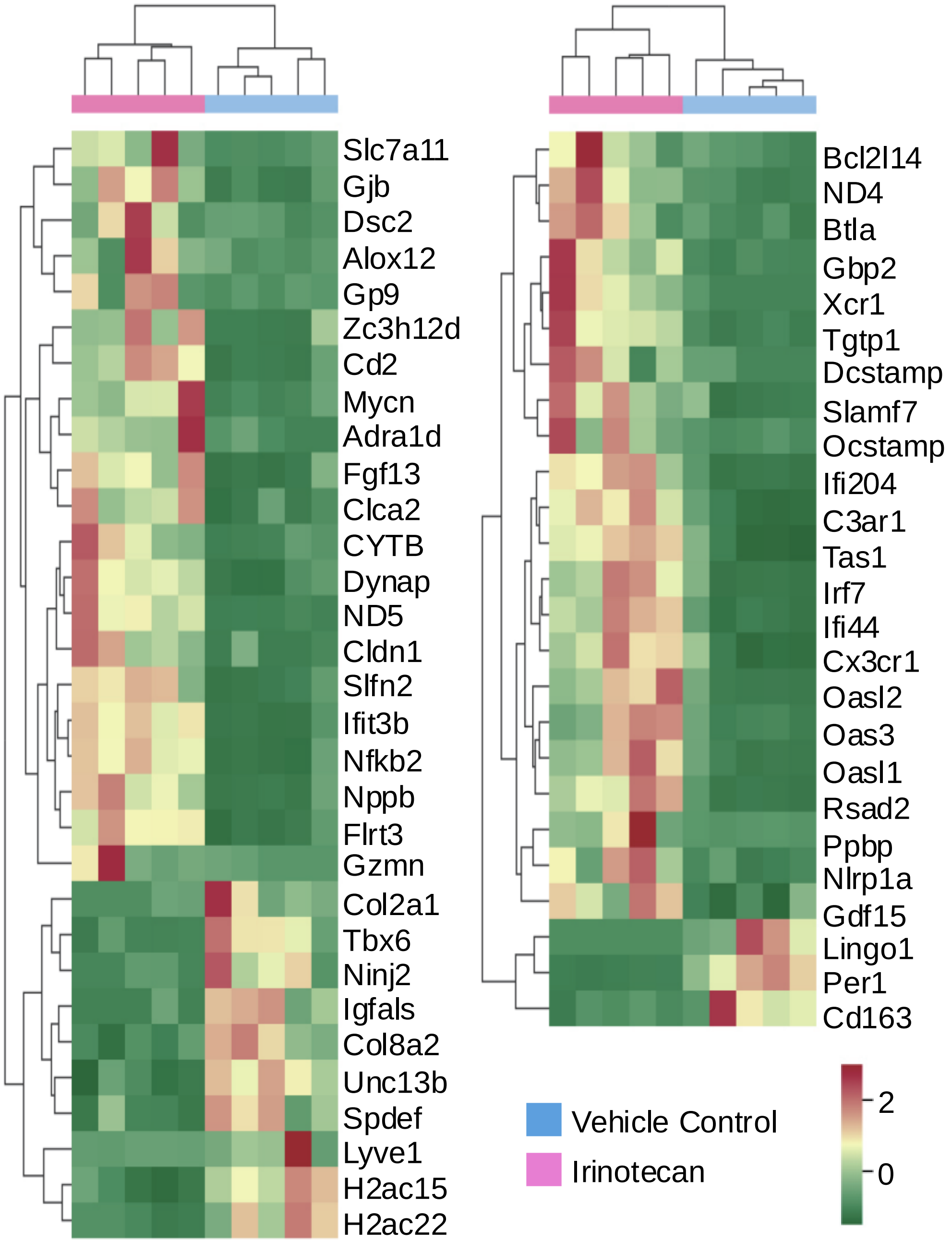

Supplementary Figure S4. Heatmap of RNA sequencing analysis of irinotecan-treated MC38 tumors. Heatmap showing differentially expressed genes involved in membrane transport and mitochondrial metabolism, among other processes in GO analyses.

Original uncropped western blot membrane figures:
All Western blots were probed using enhanced chemiluminescence and developed on X-ray films. Raw images are supplemented with hand-written molecular weight markers. Blots are corresponding to representative figures shown in main manuscript.

Figure 1A

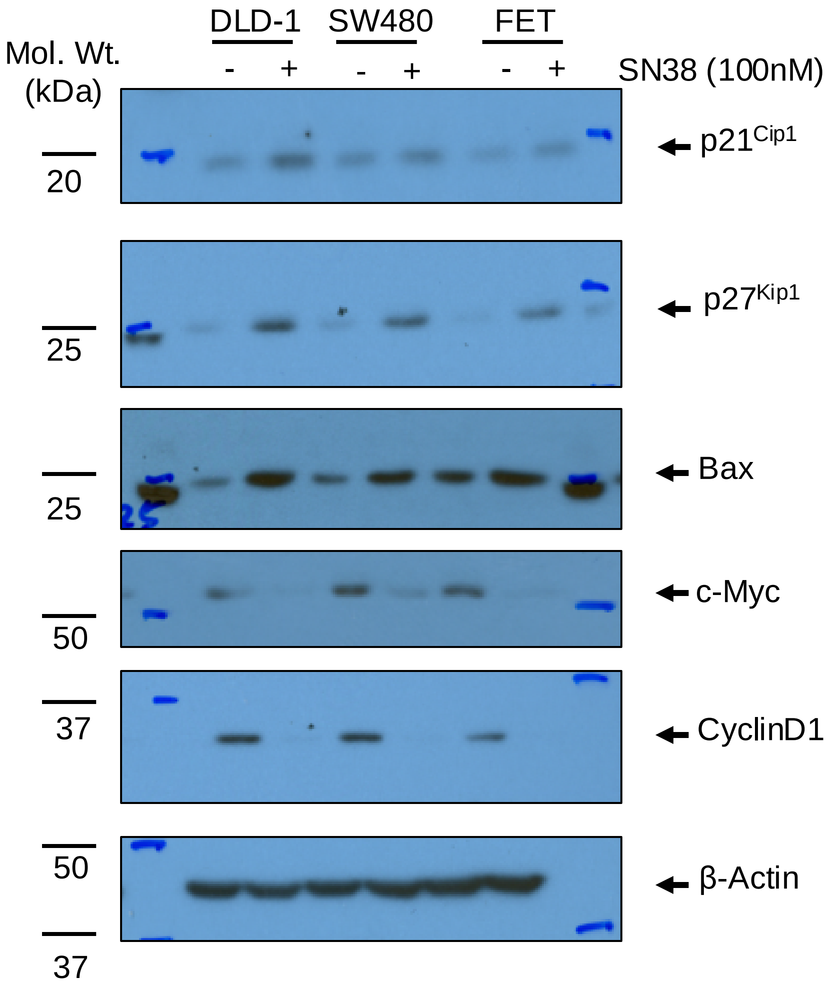

Figure 1E

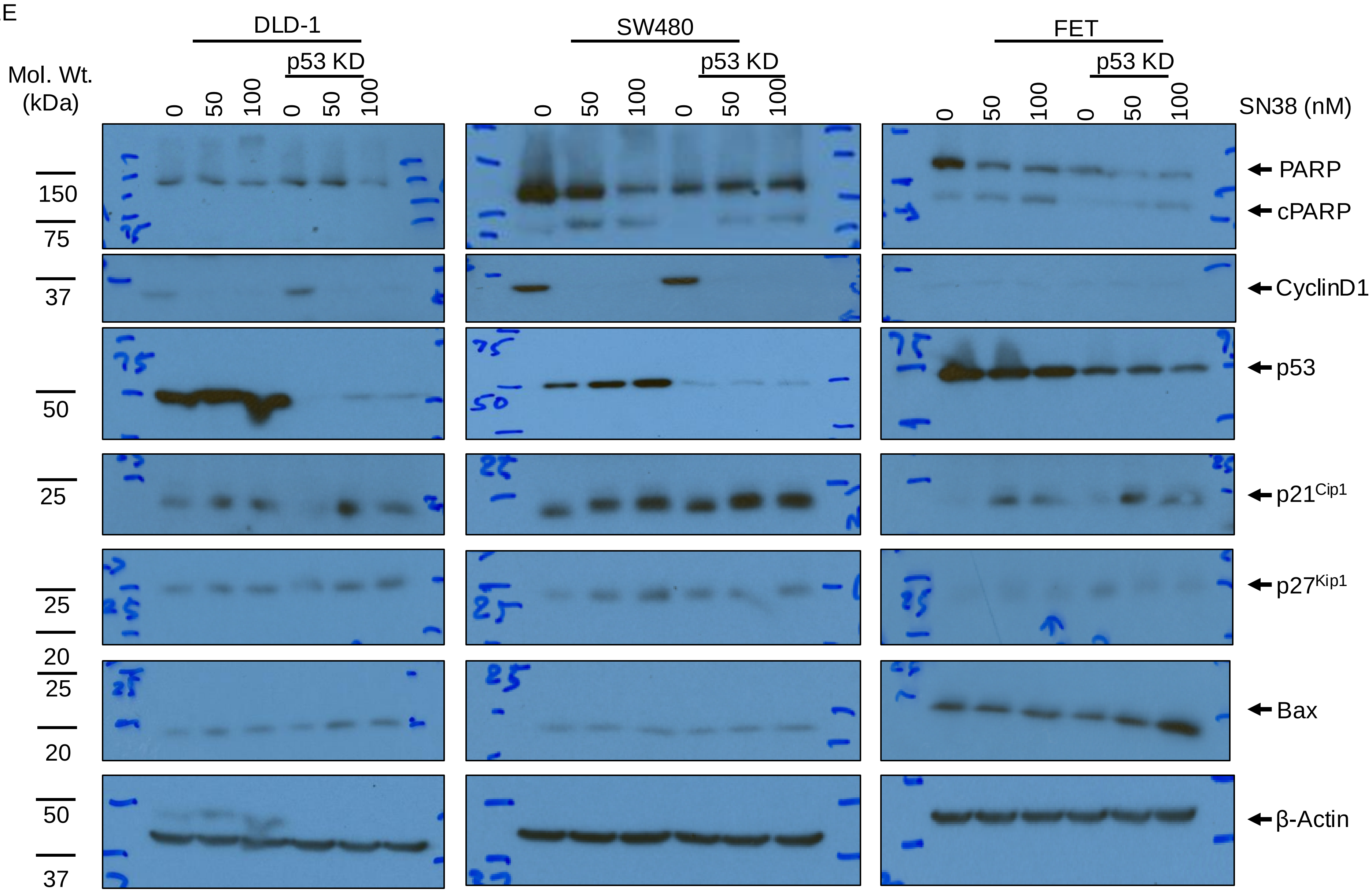

Figure 2A

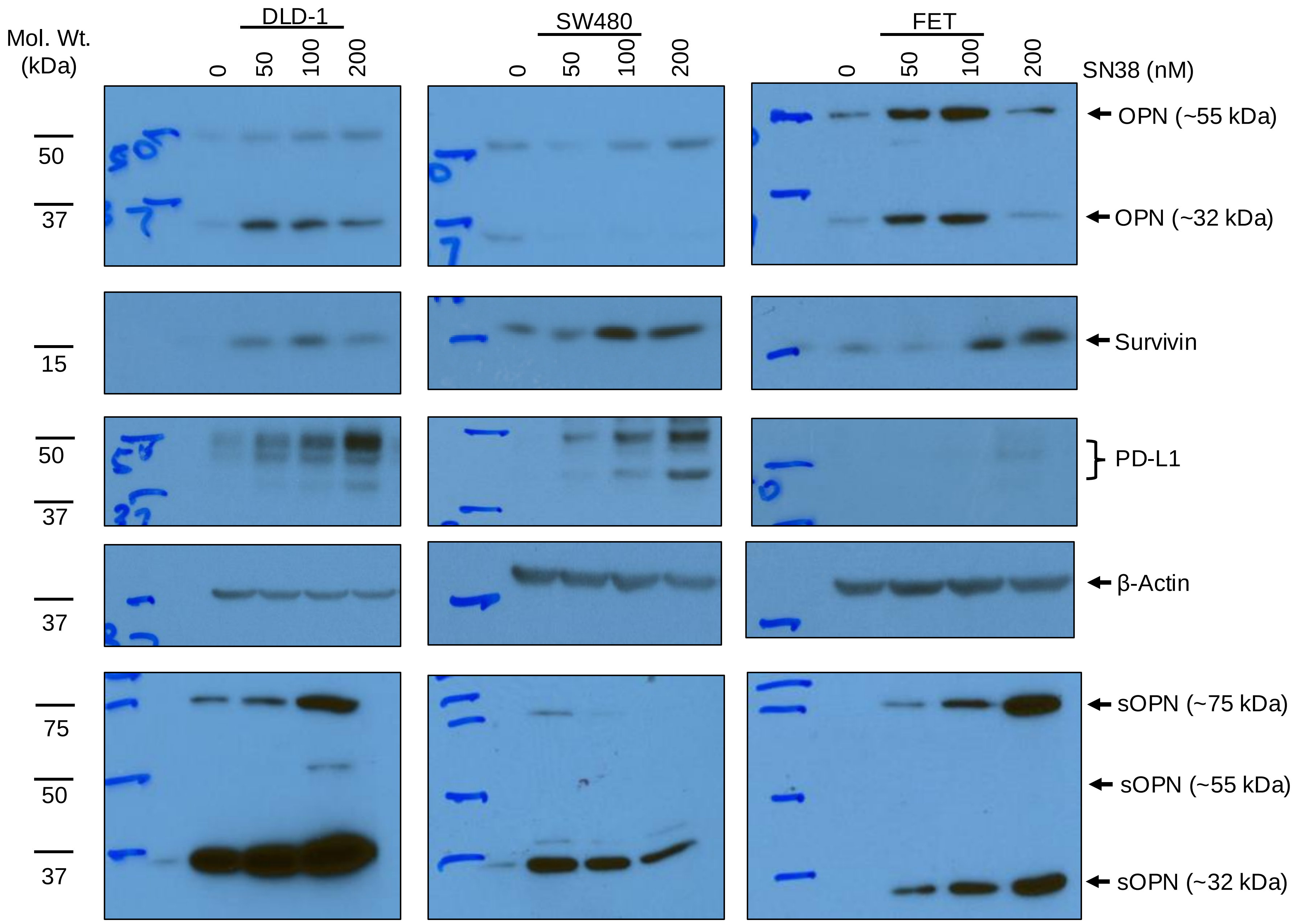

Mol. Wt.
(kDa)
DLD-1
SW480
FET
0
50
100
200
SN38 (nM)
50
37
15
50
37
37
75
50
37
OPN (~55 kDa)
OPN (~32 kDa)
Survivin
PD-L1
β-Actin
sOPN (~75 kDa)
sOPN (~55 kDa)
sOPN (~32 kDa)

Figure 2B

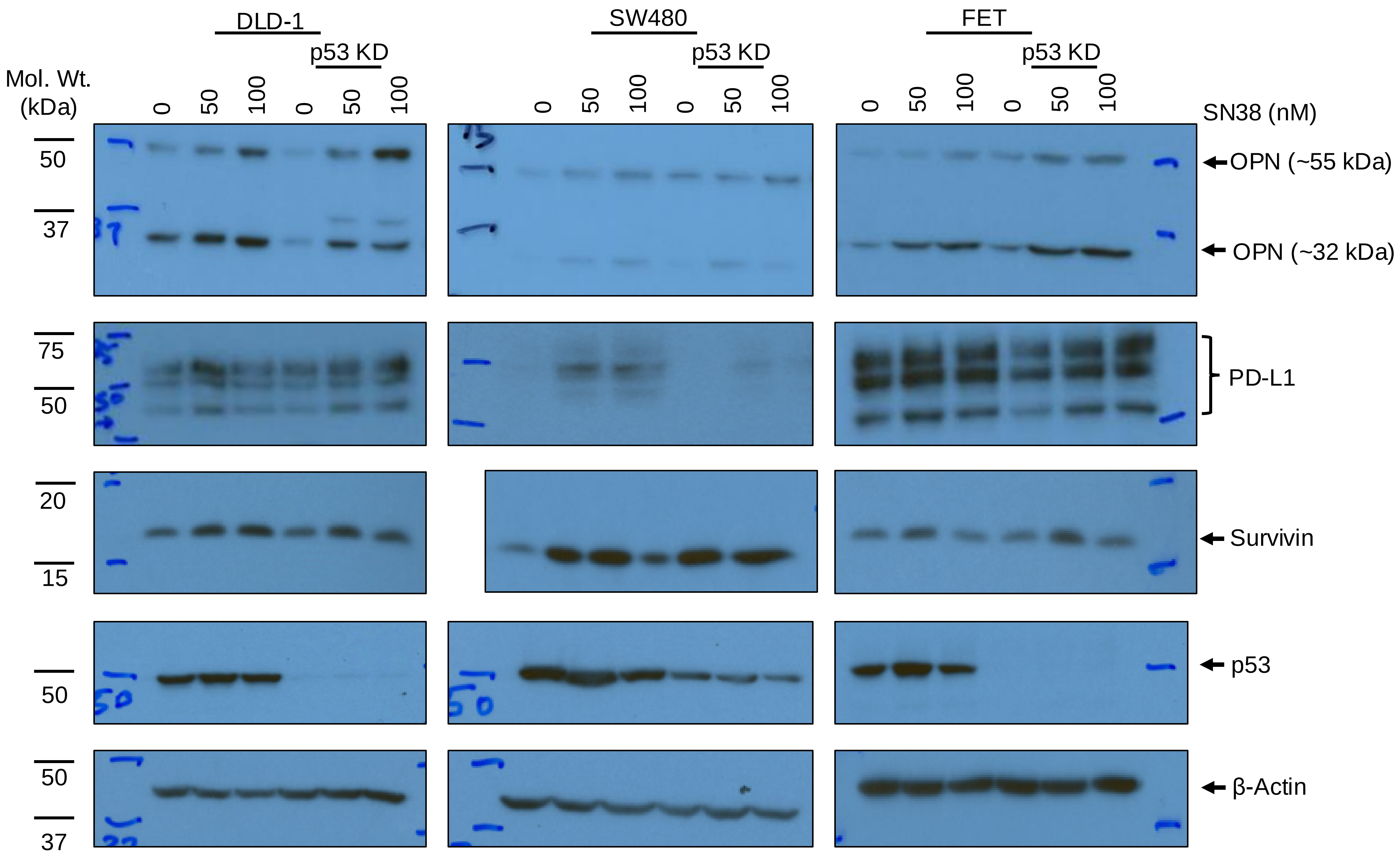

Figure 2E

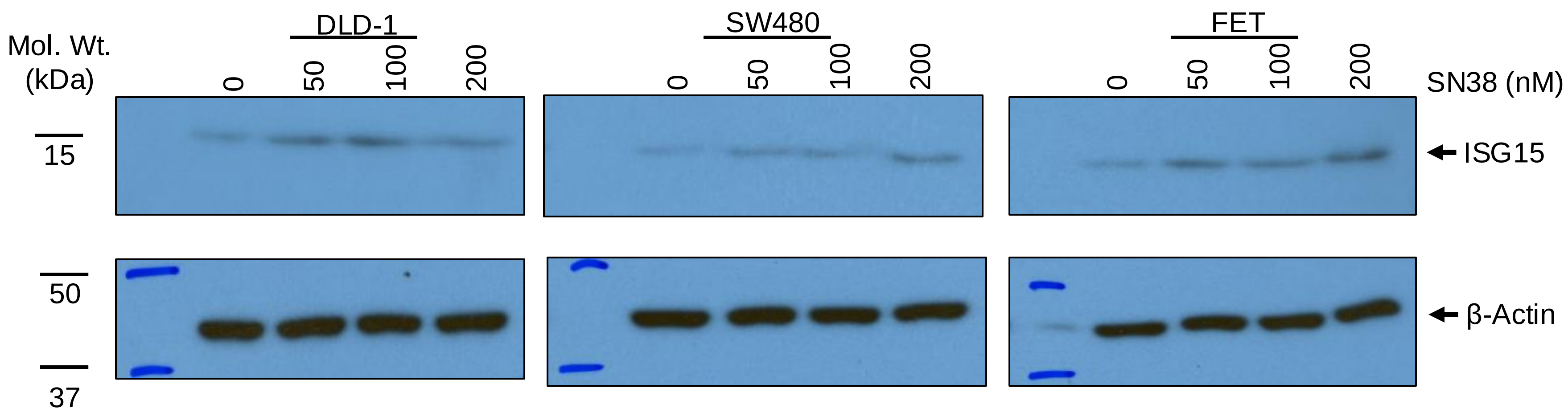

Figure 3A

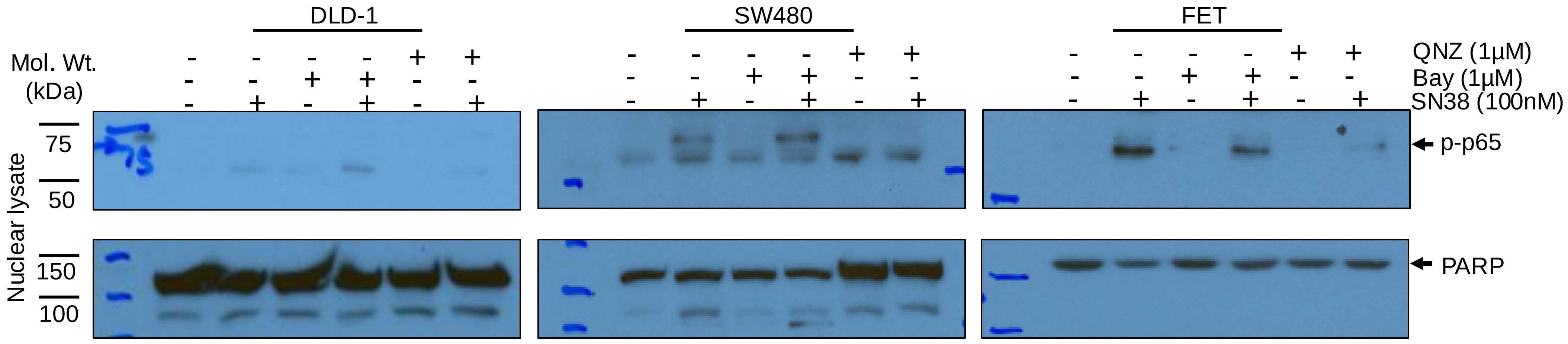

Figure 4A

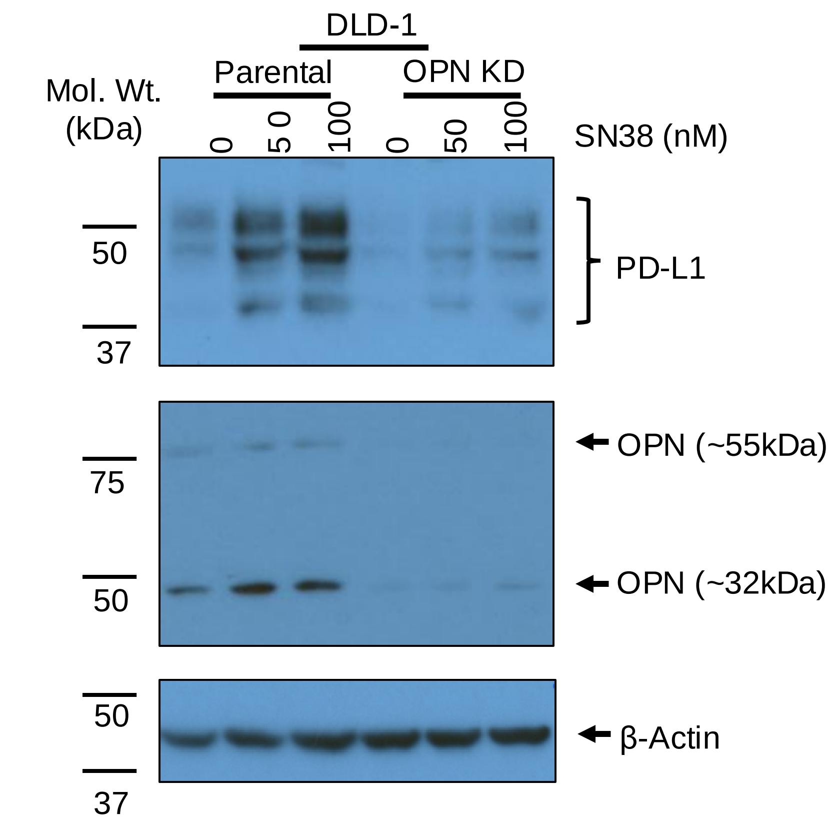

Figure 4B

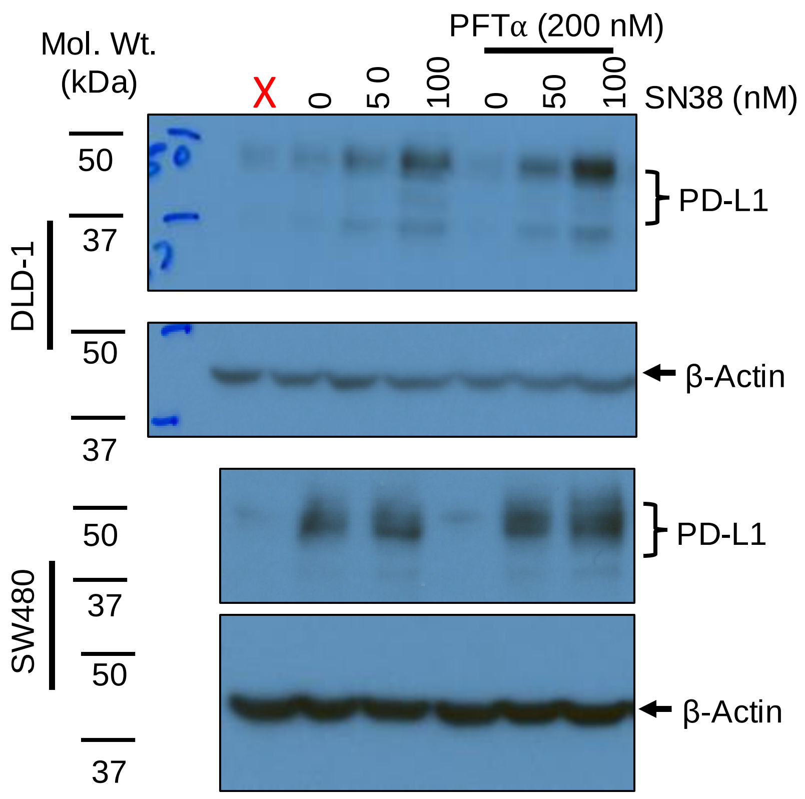

Figure 4F

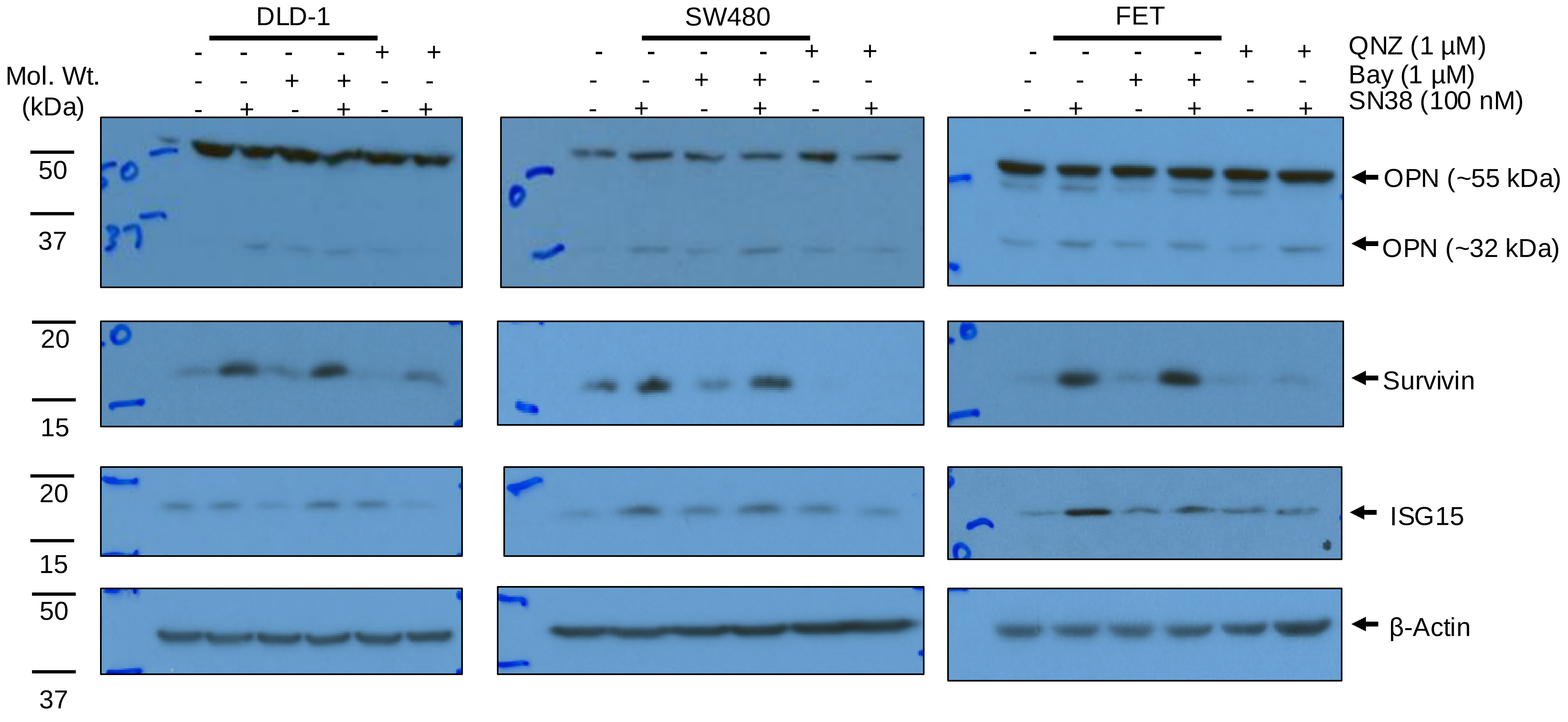

Figure 5B

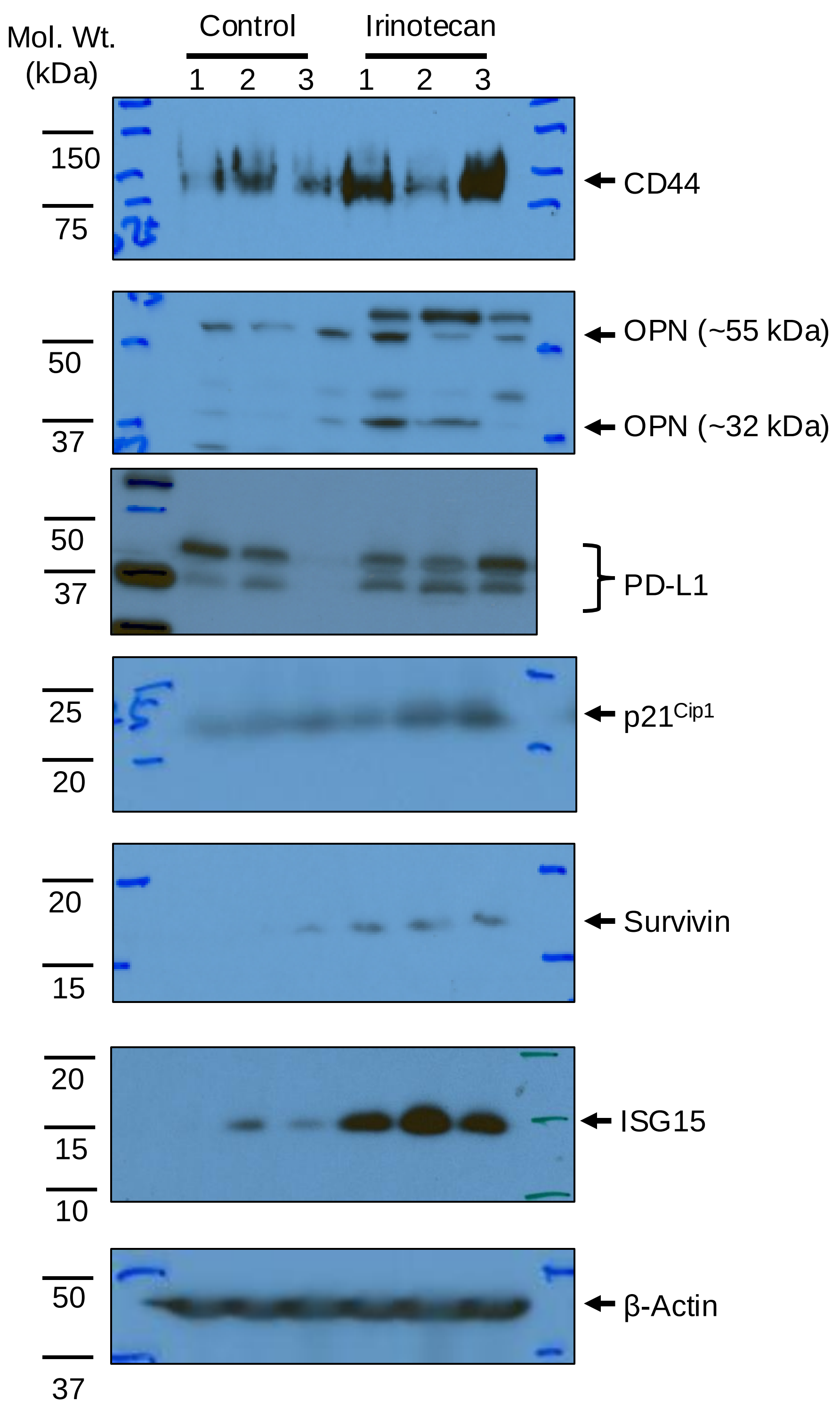
Mol. Wt. (kDa)
Control
Irinotecan
1 2 3 1 2 3
150
75
CD44
50
37
OPN (~55 kDa)
OPN (~32 kDa)
50
37
PD-L1
25
20
p21Cip1
20
15
Survivin
20
15
10
ISG15
50
37
β-Actin

Supplementary Figure S1

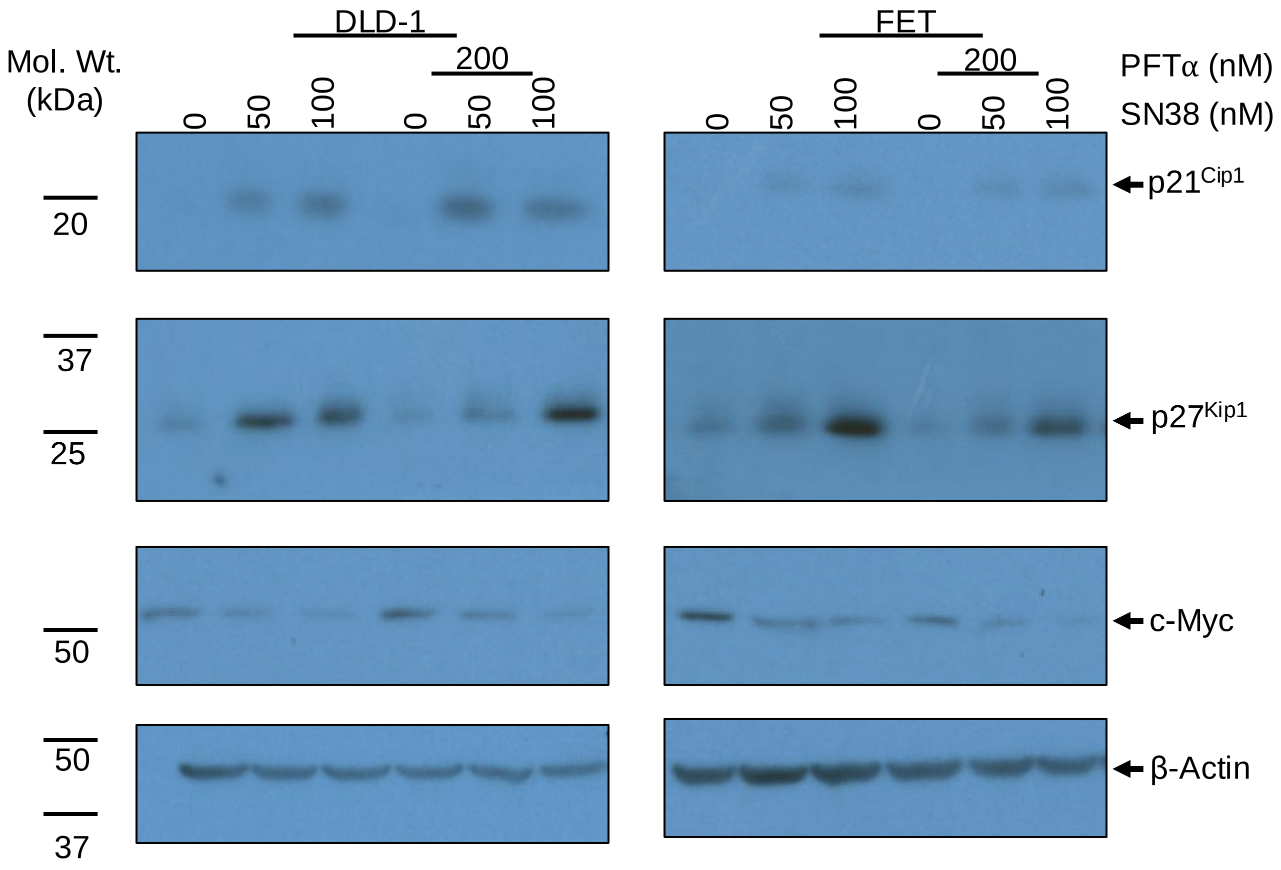

Supplementary Figure S2

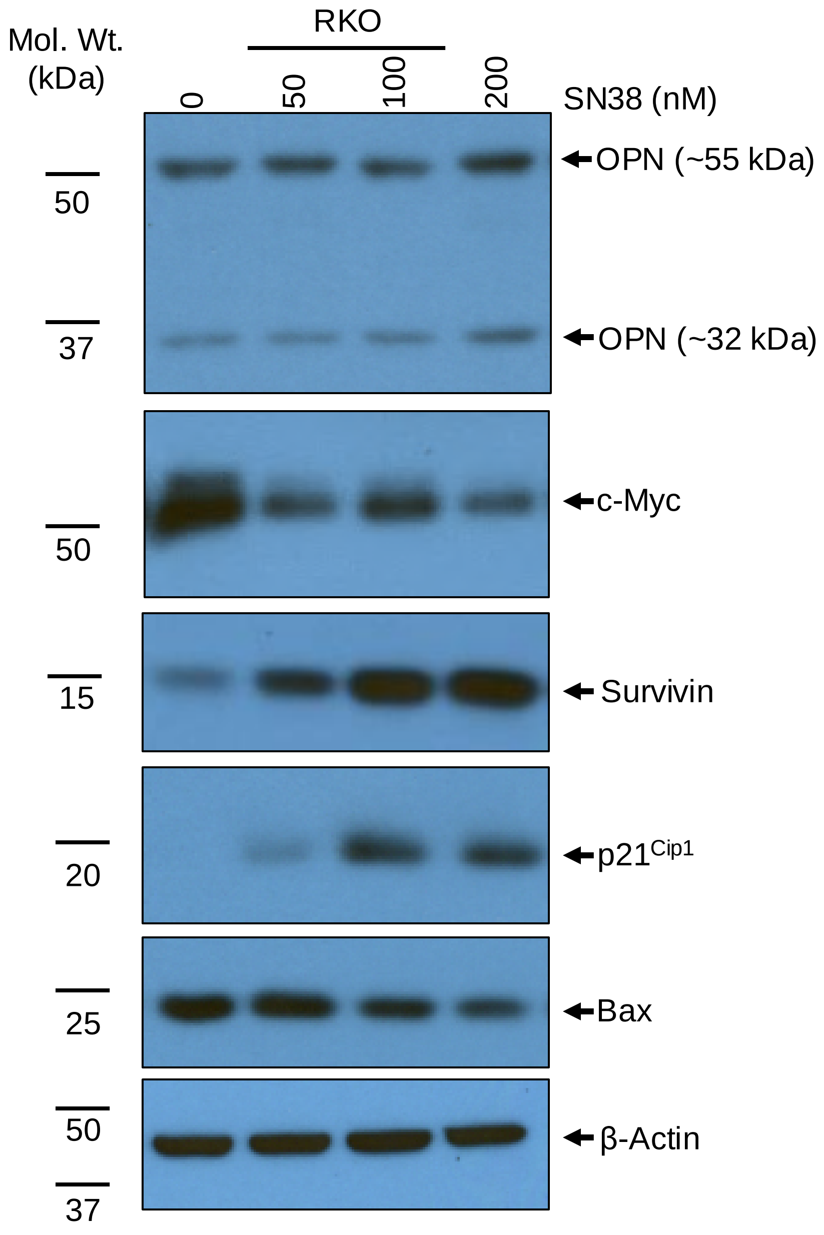

Supplement: Supplementary file 1 [file cancers-16-03491-s001.zip › cancers-3235107-supplementary.pdf]
